# A panoramic view of the genomic landscape of the genus *Streptomyces*

**DOI:** 10.1099/mgen.0.001028

**Published:** 2023-06-02

**Authors:** Marios Nikolaidis, Andrew Hesketh, Nikoletta Frangou, Dimitris Mossialos, Yves Van de Peer, Stephen G. Oliver, Grigorios D. Amoutzias

**Affiliations:** ^1^​ Bioinformatics Laboratory, Department of Biochemistry and Biotechnology, University of Thessaly, 41500 Larissa, Greece; ^2^​ School of Applied Sciences, University of Brighton, Huxley Building, Lewes Road, Brighton BN2 4GJ, UK; ^3^​ Microbial Biotechnology-Molecular Bacteriology-Virology Laboratory, Department of Biochemistry and Biotechnology, University of Thessaly, 41500 Larissa, Greece; ^4^​ Department of Plant Biotechnology and Bioinformatics, Ghent University, 9054 Ghent, Belgium; ^5^​ Center for Plant Systems Biology, VIB, 9054 Ghent, Belgium; ^6^​ Center for Microbial Ecology and Genomics, Department of Biochemistry, Genetics and Microbiology, University of Pretoria, Pretoria 0028, South Africa; ^7^​ College of Horticulture, Nanjing Agricultural University, Nanjing, 210095, PR China; ^8^​ Department of Biochemistry, University of Cambridge, Cambridge CB2 1GA, UK

**Keywords:** Streptomyces, comparative genomics, evolution, adaptation, core genome, species-specific adaptations, secondary metabolites, carbohydrate-active enzymes, TTA codon

## Abstract

We delineate the evolutionary plasticity of the ecologically and biotechnologically important genus *Streptomyces *, by analysing the genomes of 213 species. Streptomycetes genomes demonstrate high levels of internal homology, whereas the genome of their last common ancestor was already complex. Importantly, we identify the species-specific fingerprint proteins that characterize each species. Even among closely related species, we observed high interspecies variability of chromosomal protein-coding genes, species-level core genes, accessory genes and fingerprints. Notably, secondary metabolite biosynthetic gene clusters (smBGCs), carbohydrate-active enzymes (CAZymes) and protein-coding genes bearing the rare TTA codon demonstrate high intraspecies and interspecies variability, which emphasizes the need for strain-specific genomic mining. Highly conserved genes, such as those specifying genus-level core proteins, tend to occur in the central region of the chromosome, whereas those encoding proteins with evolutionarily volatile species-level fingerprints, smBGCs, CAZymes and TTA-codon-bearing genes are often found towards the ends of the linear chromosome. Thus, the chromosomal arms emerge as the part of the genome that is mainly responsible for rapid adaptation at the species and strain level. Finally, we observed a moderate, but statistically significant, correlation between the total number of CAZymes and three categories of smBGCs (siderophores, e-Polylysin and type III lanthipeptides) that are related to competition among bacteria.

## Data Summary

**Fig. 1.**
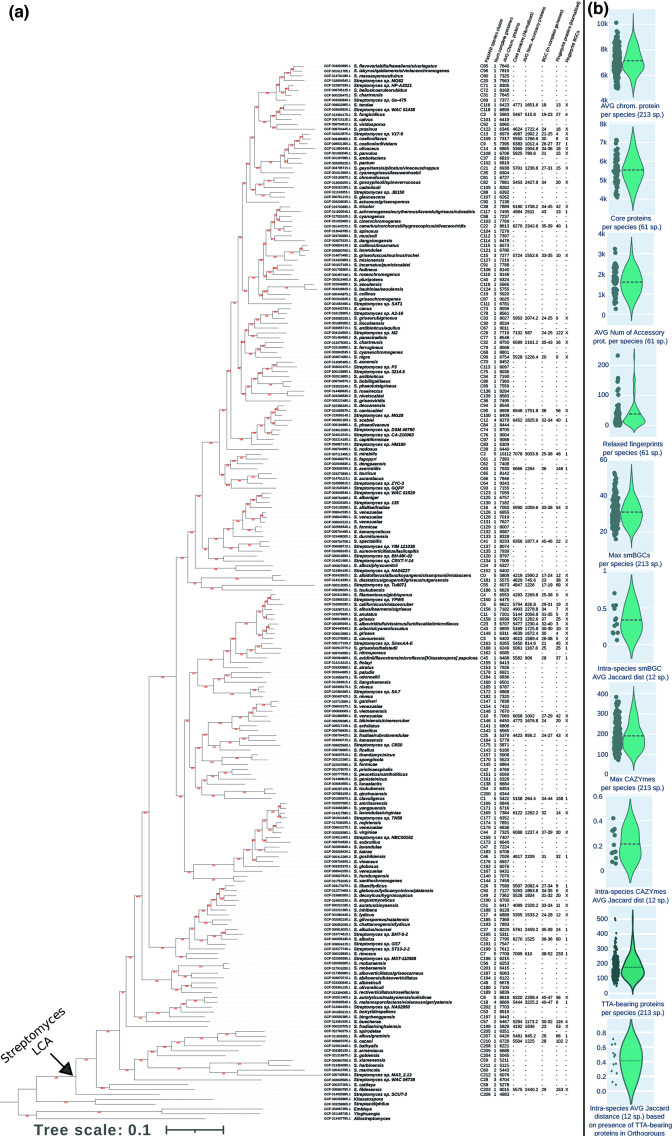
(a) The *Streptomyces* phylogenomic species tree is based on 213 high-quality *Streptomyces* genomes (one representative from each species) and five other *Streptomycetaceae* (as outgroups) and calculated from 318 core protein orthologous groups (78 205 amino acid sites) using the LG+I+F+G4 model in the IQ-Tree2 software. Next to the tree, on the first column, we show the various species names. On the second column is the FastANI cluster of the corresponding species. On the third column is the number of high-quality genomes in that species. The next six columns correspond to the average number of chromosomal proteins, the number of normalized core proteins, the average number of accessory proteins, the number of smBGCs in the complete genomes, the number of relaxed fingerprint proteins, and the number of smBGCs that are identified as fingerprints, for each species. All these species data are also summarized as a table in File S1, spreadsheet 5. (b) Violin plots that show the distribution of several genomic characteristics of the analysed *Streptomyces* species (from top to bottom): the chromosomally encoded proteins for the 213 species; the core proteins identified in each of the 61 species, with five genomes (complete and draft) each; the accessory proteins in each of the 61 species; the relaxed fingerprints for the 61 species; the maximum number of smBGCs in the high-quality genomes of each of the 213 *Streptomyces* species; intra-species smBGC average variation, based on Jaccard distance, of the 12 *Streptomyces* species that had five complete genomes each; the maximum number of CAZYmes in the high-quality genomes of each of the 213 *Streptomyces* species; intra-species CAZYme average variation (based on Jaccard distance), of the 12 *Streptomyces* species that had five complete genomes each; the number of TTA-bearing proteins in the representative high-quality genomes of each of the 213 *Streptomyces* species; and intra-species TTA-bearing proteins average conservation variation (based on Jaccard distance), of the 12 *Streptomyces* species that had five complete genomes each. The dashed line within each violin plot represents the mean value.

**Fig. 2.**
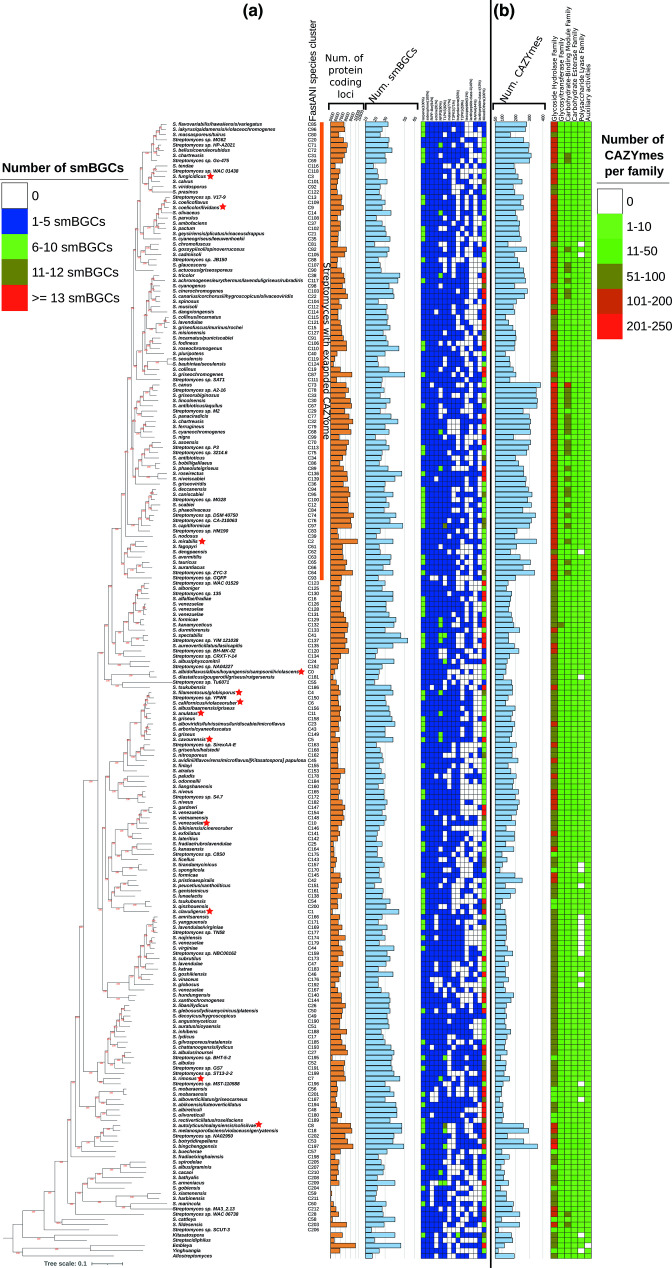
(a) Number of smBGCs in the 213 *Streptomyces* species. Only one high-quality genome with the highest number of smBGCs for each species was analysed with antiSMASH. To the left is the phylogenomic tree of the 213 species. The yellow horizontal bars represent the total number of chromosomally encoded proteins for that species representative. Next to it, the light blue horizontal bar represents the total number of smBGCs for that species representative. The smBGC heatmap only includes the most frequently found (present in at least 40% of species) smBGCs. Above each column is the type of smBGC and, in parentheses, the percentage of species that is present. (b) Number of carbohydrate-active enzymes (CAZymes) in the various species. The horizontal light blue bars represent the total number of CAZymes for that species representative. The CAZyme heatmap shows the abundance of each of the six CAZyme categories in each species. All these species data are also summarized in File S1, spreadsheet 5. The orange vertical bar to the right of the phylogenomic tree represents the *Streptomyces* lineage that has a significantly higher number of genes for these enzymes, compared to the other *Streptomyces* species. Red stars denote the 12 species that were used to calculate the intra-species heterogeneity in the number of smBGCs, CAZymes and the conservation of TTA-bearing proteins.

**Fig. 3.**
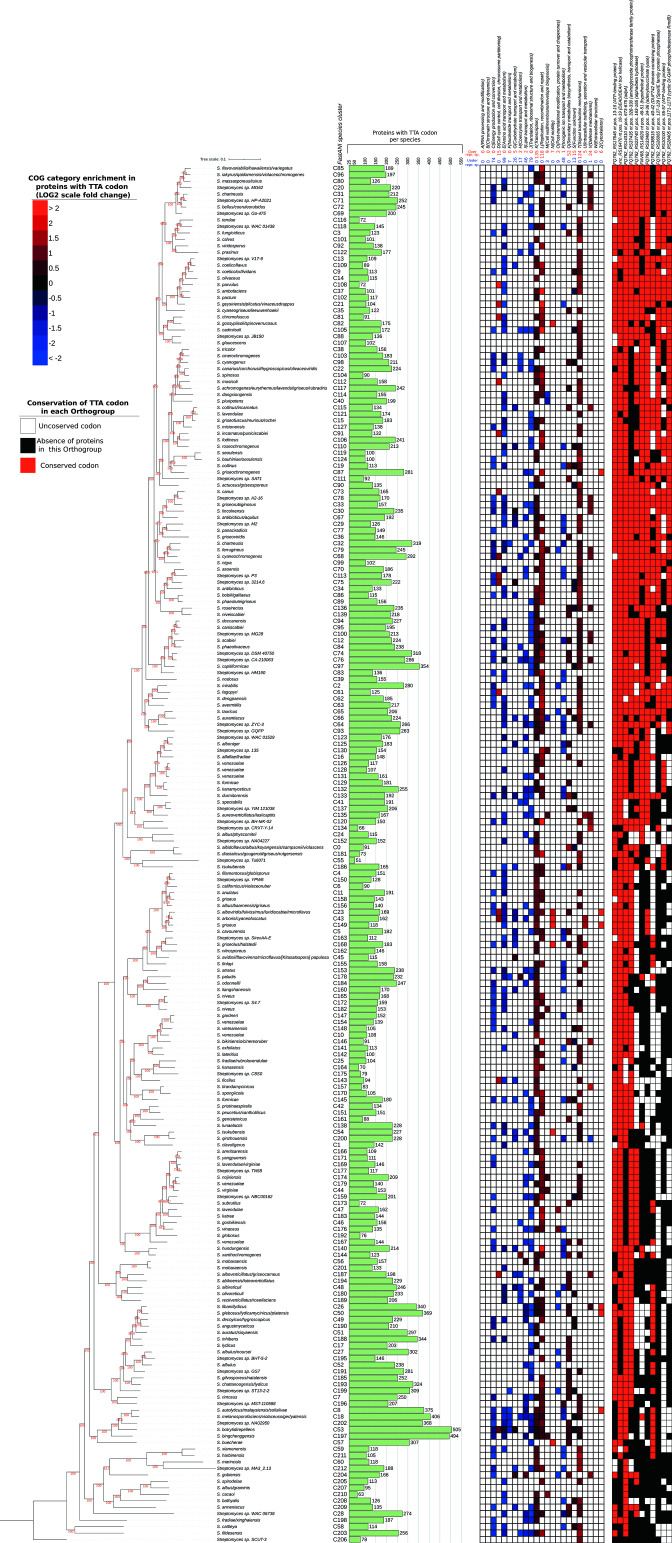
Presence of chromosomally encoded TTA-bearing ORFs in the 213 *Streptomyces* species (green bars). Only one high-quality genome for each species was analysed. To the immediate right of the barchart is a heatmap of the statistically significant enrichment of the different COG categories in TTA-bearing ORFs per species (fold-change on log_2_ scale). Over-representation in a particular category is marked with red colour, while under-representation is marked with blue colour. Above the heatmap is the total number of species in which a certain category is found to be over/under-represented. To the far right is a presence/absence matrix of the 11 TTA-codons whose position is conserved in at least 50% of the species that have the orthogroup. Presence of TTA-bearing ORFs is shown in red, while absence is shown in white. Orthogroups of TTA-bearing ORFs that are not present in a certain species are marked black. All these species data are also summarized in File S1, spreadsheet 5.

The three figures of the manuscript, one supplementary_trees zip file with the three corresponding newick phylogenomic trees and their iTOL annotation files (for Figs 1–3), one supplementary excel file with 16 worksheets, nine supplementary figures and one supplementary mp4 file are available for this article in Figshare with https://doi.org/10.6084/m9.figshare.22316791.v2 [1].

**Impact Statement**

*Streptomycetes* are aerobic, filamentous Gram-positive *Actinobacteria* that have a complex life cycle and large linear genomes. So far, the genus is known to contain >600 species which are of great ecological and biotechnological importance, being major biomass degraders and producers of many secondary metabolites that have antibacterial, antifungal, antiparasitic and even anticancer activities. These organisms have great potential for the development of new biotechnological processes (i.e. as hosts for the production of compounds) using the techniques of synthetic biology. Genome mining techniques are key to such developments and, to exploit them, it is essential that *Streptomyces* species are clearly delineated and that the core and accessory components of each species’ genome are defined since that will guide the genome reduction strategies that are the prelude to wholesale genome engineering. This is, to date, the most comprehensive large-scale comparative analysis of the genomic plasticity of the streptomycetes at the genus and species level, based on 742 genomes from 213 *Streptomyces* species. Our analyses showed a high proportion of regulatory protein-coding gene content of the *Streptomyces* species, high volatility of the species-level core/accessory proteins, smBGCs, CAZymes, TTA-bearing ORFs, a high proportion of paraloges, and an already complex genome of the *Streptomycetes* last common ancestor. Intriguingly, the total number of CAZymes correlates moderately with three categories of smBGCs (siderophores, e-Polylysin and type III lanthipeptides), that are related to competition among bacteria. Our analyses further showed that the conserved core components of the genome are located in the central region of the linear chromosome, proximal to the replication origin. In contrast, accessory (often species-specific) components are located distal to the replication origin on the chromosome arms. It is to these distal regions that genome reduction efforts should be directed. The chromosome arms are also the regions that may be mined for genes encoding biotechnologically valuable proteins involved in carbohydrate metabolism, secondary metabolism and gene regulation. Finally the evolutionary volatility of the chromosome arms may explain the remarkably rapid adaptability of this genus to very diverse environments.

## Introduction

*Streptomycetes* are aerobic, filamentous Gram-positive *Actinobacteria* that are found mostly in soil and form complexes of differentiated vegetative and aerial hyphae [2]. They are capable of decomposing insoluble organic remains of fungi, plants and other organisms using a large array of hydrolytic exoenzymes [3], while at the same time, they produce many secondary metabolites whose antibiotic activity inhibits the growth of other competitors. Thus, *Streptomycetes* are of great ecological and biotechnological importance, being major biomass degraders, and producers of numerous secondary metabolites that have antibacterial, antifungal, antiparasitic and even anticancer activity [4–6]. Organisms showing high levels of antimicrobial resistance (AMR) are leading causes of death around the world [7], thus making *Streptomyces* research a priority area.

The genomes of *Streptomycetes* are distinctive among the bacteria, since they comprise a large linear chromosome with terminal inverted repeats and a size that ranges from 6 to 12 Mb, encoding 5300 to 11 000 proteins [8–10]. A typical bacterial genome has a size of 5 Mb and encodes approximately 5000 proteins on a circular chromosome [11]. The large genome size of *Streptomycetes* is the result of horizontal gene transfer (HGT) [12], gene duplications [5, 13] and recombination [14, 15], and its evolutionary path has been partly attributed to its complex life cycle, which includes several developmental stages [13].

*Streptomyces* is an ancient genus, with well over 600 species identified so far (http://www.bacterio.net/streptomyces.html) [16] belonging to the phylum *Actinobacteria* [17]. The genus is estimated to have emerged between 440–380 million years ago, during the period that plants colonized the land [12, 18]. Phylogenetic/phylogenomic analyses have delineated the relationships among species of the genus *Streptomyces *, as well as the family *Streptomycetaceae* and even the phylum *Actinobacteria* [17, 19–23]. Several studies have focused on genomic mining and the evolution of the *Streptomycetes* secondary metabolite biosynthetic gene clusters (smBGCs) [8, 16, 24–32]. This modern approach is very useful for the discovery of novel secondary metabolites, because the majority of smBGCs are inactive under laboratory conditions [27]. Furthermore, *Streptomycetes* demonstrate a distinctive method of regulating genes involved in secondary metabolism and development, by incorporating a rare TTA-codon whose tRNA is encoded by the *bldA* gene [33–35].

These unique features of the genome and molecular biology of *Streptomycetes* have great implications for synthetic biology and biotechnology. Several studies have generated extensive deletions of accessory genes, many of them smBGCs, in order to reduce the size of *Streptomyces* genomes to generate a ‘chassis’ for the heterologous expression of secondary metabolites, and other products, with high yield [27, 36–41]. However, for genome reduction to succeed, it is imperative to determine which are the core and accessory components of each species’ genome. Thus, several studies have analysed the core and accessory genomes of the genus *Streptomyces* [8, 9, 28, 42]. In addition, knowledge of so-called ‘species-fingerprint’ genes is very important. We have previously defined ‘fingerprints’ as any orthologous gene of a given bacterial species that is present in all members of that species, while being absent from all other species of the genus [43, 44]. Such fingerprints are expected to be important for the particular phenotypic characteristics and adaptations of a given species. Furthermore, they may also be important targets for the future development of highly specific drugs or vaccines.

The goal of this study is to understand the evolutionary genomic plasticity of the various members of the genus *Streptomyces* by comparative analysis of a large number of both high-quality (i.e. complete/chromosome assembly level) and draft (scaffold/contig assembly level) publicly available genome sequences. To this end, we have performed a series of large-scale comparative analyses to identify (i) the ecological diversity of the various species and strains, (ii) the core proteins of the genus, (iii) the core proteins of each of its species, (iv) the species-specific fingerprint-proteins for those species for which sufficient numbers of genome sequences are available to permit meaningful analysis, (v) the patterns of homology and genomic expansion in the various lineages of the genus, and (vi) the distribution and diversity of the smBGCs, the carbohydrate-active enzymes and the rare TTA-codon-bearing proteins at the genus and species level. All the above will also help guide future efforts to design more efficient synthetic *Streptomyces* genomes.

## Methods

Publicly available actinobacterial and *Streptomyces* genomes were retrieved from the NCBI RefSeq database (latest download on September 2022; list of genomes available in Excel File S1, available in the online version of this article, sheets 1 and 2). The core proteomes and the fingerprints of the various lineages (genus/species) were identified with a series of python scripts that were developed previously by our group [43, 44]. Due to the biased sequencing of the various lineages/species, we generated a normalized dataset, where at most five genomes were selected for each species (if available). This normalization allows for a more meaningful comparison of lineages/species that do not have the same sampling depth. For certain analyses, we identified orthologous groups (orthogroups) based on Orthofinder (default parameters) [45]. Phylogenomic analyses were performed with Muscle [46], G-blocks [47], IQTree2 [48], Treedyn [49] and iTOL [50], whereas branch support was estimated with the approximate likelihood ratio test (aLRT) [51]. Specifically, the phylogenomic tree depicted in Figs 1 and 2 includes 213 *Streptomyces* representative genomes (one for each species) and five *Streptomycetaceae* genomes as outgroups. This phylogenomic tree was generated from a set of 318 orthologous protein groups that were present in all the 218 genomes. Each of the 318 reference proteins and their corresponding orthologues were aligned using muscle and all individual alignments were then concatenated into a super-alignment. This super-alignment, after filtering with G-blocks (default parameters), consisted of 78 205 amino acid sites. This alignment was fed to IQTRee2 and tree calculation was performed using the LG+I+F+G4 model and branch support was assessed using aLRT. The phylogenomic tree of Fig. 3 was based on the 466 orthologous protein groups (see Excel File S1, sheet 3) that were present in all 213 *Streptomyces* genomes (one representative from each species) and the corresponding super-alignment, after filtering, consists of 117 681 amino acid sites. The LG+I+F+G4 model was used for this tree and branch support was assessed using aLRT. Both phylogenomic trees (of 218 and 213 species), their filtered super-alignments and their relevant iTOL annotation files for each of the three figures are available in supplementary_trees.zip file (in Figshare).

Species demarcation was based on average nucleotide identities (ANI values) with FastANI, based on the 95 % species cut-off [52]. Subsequently, the species clusters were generated with the Markov Cluster (MCL) algorithm [53], as described previously [43].

We estimated the theoretical total number of species of this genus, by curve fitting the saturation curve of the cumulative number of sequenced genomes (*x*-axis) versus the estimated number of species (*y*-axis). This saturation curve is best approximated by an exponential recovery function (y=a*(1−*e* ^(*−x*/*b*)^)), as in [54], where *x* is the cumulative number of genomes sequenced and *y* the number of species. The curve function parameters (*a* and *b*) were calculated/fitted using the solver add-in of Microsoft Excel with the Generalized Reduced Gradient (GRG) non-linear method by minimizing the sum of squared errors between the observed and the theoretical (as calculated from the curve function) number of species. Towards this end, we used all the available 477 complete and 2291 draft (scaffold and contigs) *Streptomyces* genomes from NCBI RefSeq. In total, the 2768 genomes clustered in 961 species. These data are available in File S1, sheet 4.

Proteins were assigned to functional categories using the eggNOG mapper software version 2 [55], based on the EGGNOG database version 5 [56], KEGG [57] and COG [58]. The statistical significance of over/under-representation of the COG functional categories in the various analyses was performed with the hypergeometric test of the python Scipy package [59]. The results are summarized in File S1, sheets 7 and 9. The identification of smBGCs was performed only for high-quality genomes and not for scaffold/contig assembly-level genomes, in order to ensure cluster integrity, using the antiSMASH v6 software [60]. Intra-species variability (presence/absence) of highly similar homologues for each cluster was calculated using the Jaccard distance from the Scipy package [59]. Carbohydrate-active enzyme (CAZyme) identification/annotation for the analysed genomes was performed with the DBCan2 tool [61] based on the CAZy database [62]. Identification of rare TTA-codon-bearing ORFs and conservation in the various orthogroups was performed with custom Python scripts.

Homologues within each of the analysed genomes were identified with the DIAMOND software (sensitive mode). Genome-scale dotplots of each of the genomes against itself were created with the D-genies software (default parameters) [63] and the Minimap2 v2.24 aligner (many repeats option) [64]. The presence/absence of ancient homology blocks was investigated with pyCircos (https://github.com/ponnhide/pyCircos) and custom scripts.

We estimated the hierarchical orthogroups and duplication/loss profiles of various *Streptomyces* ancestors with OMA [65], based on a representative sample of the genus *Streptomyces* (20 species) along with five *Streptomycetaceae* genomes as outgroups. The gene evolution profile (gene duplications, losses, gains, retentions) on the various *Streptomyces* ancestral nodes was calculated and visualized using the pyHAM module [66].

## Results and discussion

### Phylogenomic analysis, species demarcation and misannotations of the genus *Streptomyces*

Phylogenomic analysis of the genus *Streptomyces *, combined with the ANI analysis (≥95 % identity cut-off for species demarcation) identified 213 species, of which only three were not monophyletic and thus dubious. Each of the 213 species contains at least one high-quality genome. The phylogenomic tree of the 213 species, based on one representative genome per species, is shown in Fig. 1. A more complete phylogenomic tree, based on 742 genomes, is shown in Fig. S1.

Misannotation, in terms of species names, is frequently observed in various bacteria [43, 44], including the genus *Streptomyces* [67–71]. This is most probably a result of earlier typing methods with less resolution than today’s phylogenomic methods. It is very important to identify such misannotations in order to avoid artefacts in the estimation of the core genome and the fingerprints of each species. We identified several misannotations, where strains with the same species name had been ascribed to different monophyletic species clusters (based on phylogenomics and ANI values). Such examples are highlighted in red on the phylogenomic tree of Fig. S1. We observed that 44 monophyletic and three non-monophyletic species clusters (based on phylogenomics and ANI values) included genomes with at least two different species names (see Figs 1 and S1).

We further analysed 2768 sequenced *Streptomyces* genomes (complete, scaffold and contigs) and, based on their ANI values (determined with FastANI), we clustered them into 961 species. Based on curve-fitting of the saturation curve (exponential recovery) of these 2768 sequenced genomes versus the number of species, we estimated the theoretical total number of *Streptomyces* species to be approximately 1600 (see File S1, spreadsheet 4). However, this analysis should be treated with caution because the analysed genomes are not randomly sampled, but are mainly based on focused genome projects. A more accurate estimation would be obtained only by unbiased large-scale metagenomic projects that would sample many diverse environments.

### Ecological diversity and rapid adaptation of the *Streptomyces* species and strains

Based on GenBank annotation, the isolation source was available for 514 of the 742 *Streptomyces* genomes that were assigned to 213 species. The vast majority (397/514: 77%) were isolated from terrestrial environments (i.e. soil, land plants, land animals) whereas 92 (18 %) were isolated from marine environments (i.e. deep sea, marine plants, marine animals). We also observed that strains of the same species were identified in very diverse environments. Notably, strains of the C0 species cluster were isolated from marine environments, marine animals, soil, faeces and intestinal tract (see Fig. S1). In addition, 31 species had both marine and terrestrially sampled sequenced strains (complete or draft genomes). Interestingly, different *Streptomyces* evolutionary lineages were found in the same environment or type of host, demonstrating the ability of the genus for rapid adaptation to very diverse environments. For example, the strains that were populating marine environments or insects did not form monophyletic groups and thus were not distinct from their terrestrial relatives. These findings indicate that the genus *Streptomyces* is likely to display a high level of genome plasticity that involves those elements which play a key role in rapid adaptations in diverse environments, such as genes for secondary metabolites as well as those for biomass-decomposing enzymes and their regulators.

### Functional categories of the *Streptomycetes* proteomes

We compared the protein content, in terms of functional categories, of the 213 members (one from each species) of the genus *Streptomyces* against that of 192 representatives (one from each genus) of the phylum *Actinobacteria* and 55 representatives (one from each species) of the genus *Bacillus *. The functional categories (as percentage of the total number of chromosomal proteins) of Transcription, Lipid transport and metabolism, Signal transduction, and Secondary metabolism were significantly elevated (*t*-test *P*<0.05) in the genus *Streptomyces *, compared to the entire phylum *Actinobacteria* and the genus *Bacillus* (see Figs S2 and S3). It was previously shown that genes involved in Transcription were over-represented in the *Streptomyces* pangenome [72]. Another recent study further demonstrated that many of the Transcription Factors are strain-specific [8]. Intriguingly, we observed that a significant proportion (~15 %) of the *Streptomyces* proteome is involved in Transcription and Signal Transduction. This proportion is significantly higher than that observed in many (though not all) *Actinobacteria *, as well as in other Gram-positive bacteria, such as the *Firmicutes* (e.g. *Bacillus *). This reflects the regulatory complexity needed for organisms that demonstrate multicellular development with vegetative and aerial hyphae, and with division of labour [2]. Indeed, regulatory complexity, especially in terms of Transcription Factors, has been linked to the observed complexity of cellular types in animals and bacteria [73–75]. As organisms become more complex, a higher fraction of their genome encodes regulatory proteins. In addition, the Transcription Factor and Signal Transduction families have a higher probability of being retained, following duplications in eukaryotes [76, 77] and prokaryotes [78].

Next, we integrated the protein content of the 192 representatives of the entire phylum *Actinobacteria *, together with their phylogeny, in order to understand how this significant expansion of these four functional categories in *Streptomycetes* was shared with some, though not all, other *Actinobacteria *. Our analysis indicates that the occurrence of genes in these functional categories probably expanded within the genomes of the class *Actinomycetia* and were subsequently reduced secondarily in various sub-lineages (of *Actinomycetia *; see Fig. S3). Thus, this regulatory complexity is not a unique feature of the genus *Streptomyces* but, rather, a common feature of several genera of the class *Actinomycetia *.

### *Streptomyces* genomes have expanded mostly via ancient duplication events

Although a significant proportion (28–47 %; average: 37 %) of the chromosomal proteomes of *Streptomyces* species are singletons (DIAMOND e-value cut-off: 1e-5), evidently the majority have homologes (either paralogues or xenologues) within their genomes. This proportion of singletons in the genus *Streptomyces* is significantly lower than that observed in the *Actinobacteria* as a whole. A previous analysis of five *Streptomycete* genomes revealed high numbers of gene duplicates, where duplications of both single genes or blocks of genes play an important role [5].

A significant proportion (9–35 %; average: 14 %; sd ±3%) within each of the 213 *Streptomycete* proteomes were close (or moderately close) homologues, i.e. >50 % shared amino acid sequence identity. The highest percentage (35 %) of chromosomal proteins being homologues was observed in *Streptomyces* sp. DSM 40750 (FastANI species cluster C74). A distribution of the percentages of protein-encoding homologes observed in the high-quality genomes of *Streptomyces* spp., based on five amino acid sequence identity bins (51–60, 61–70, 71–80, 81–90, 91–100 %) is shown in Fig. S4 and File S1, sheet 6.

We observed that there are significantly more highly divergent homologues than highly similar homologues. However, we also observed a few notable exceptions, where some species (eight outliers based on Z-score) underwent a recent and very significant genome expansion due to block duplications at the chromosomal arms. The lengths of such duplicated regions range from 0.203 to 1.03 Mb (average: 0.447 Mb). The most notable recent expansion belongs to *Streptomyces* sp. DSM 40750 (FastANI cluster C74). More detailed information about these eight species may be found in Figs 1, S4 and S5 and File S1, sheet 6. We also observed several very distinct cases where sister species had very different percentages of homologues. This is a clear demonstration of the dynamic nature of the *Streptomyces* genome.

Next, we wanted to understand whether this distribution of the percentage of homologues of the 213 *Streptomyces* species was similar or different to that of other *Actinobacteria *, as well as other well-studied Gram-positive bacteria, such as *Bacillus* species. At the 51–60, 61–70 and 71–80 % amino acid identity bins, *Streptomycetes* demonstrate a significantly higher percentage of chromosomal proteins being homologes, compared to the other taxa (Wilcoxon test *P*<0.05) (see Fig. S4). However, such a significant difference is not observed at the 81–90 and 91–100 % amino acid identity bins. This pattern may have arisen from more extensive ancient gene duplication events, from more HGT events and/or from higher mutation rates of paralogues/xenologues, compared to other *Actinobacteria *. While HGT is a common phenomenon in *Bacteria* [15, 79, 80], a previous study estimated that in *Streptomycetes*, only 10 genes were acquired and retained through HGT every 1 million years, arguing that the effect of HGT was previously overestimated for this particular genus [12].

In order to better understand the above observations, we subsequently estimated the gene retention/duplication/gain/loss profile of the protein-coding genes in the various *Streptomyces* ancestors with OMA and pyHAM [65, 66], based on 20 representative *Streptomycetes* and five other *Streptomycetaceae* as outgroups. This analysis suggests that the last common ancestor (LCA) of the genus *Streptomyces* already possessed a complex genome, estimated at 11 408 protein-coding genes. Subsequently, extensive gene losses as well as duplications/gains occurred in the various lineages (see Fig. S6). We further investigated whether such a complex ancestral genome of the *Streptomyces* LCA could have been the result of an ancient duplication, of the whole genome or a large block of genes, in some *Actinobacterial* ancestor. Thus, we visualized the chromosomal localization of each of the genus-level 90 % soft-core proteins and we connected with an arc their closest genus-level 90 % soft-core homologue in the *S. coelicolor* genome, using pyCircos (see mp4 File S10). However, we did not observe any such ancient large-block duplication event. It is common in bacteria that paralogues are mainly generated via small-scale tandem or operon duplications [78].

### Core proteome of the genus *Streptomyces*

The 213 *Streptomycete* chromosomes (one representative from each species) have a consistently high GC content, 68.8–74.7 % (average: 71.7 %). The size of the chromosome varies from 5.7 to 12.1 Mb (average: 8.5 Mb). Accordingly, the number of chromosomally encoded proteins varies from 4983 to 10 112 (average: 7130; see violin plots of Fig. 1). Based on our previously published computational protocol [44], the 100 % hard-core, and 95 and 90% soft-core proteomes of the genus comprise 137, 1992 and 2476 proteins respectively. This analysis was based on 61 species with five genomes each, and 152 species with fewer than five genomes each (177 high- and 129 draft-quality genomes). We also estimated the core proteome of the genus based on Orthofinder [45] and one representative genome from each of the 213 species. In this latter analysis, the 100 % hard-core, and 95 and 90% soft-core proteomes of the genus comprise 601, 2367 and 2804 orthogroups respectively. Thus, these two different methods and datasets produce generally comparable and consistent results. The *Streptomyces* pangenome was previously characterized as open, with many new genes being added as more genomes are sequenced [8, 9, 12, 28]. This is probably due to it being an ancient genus with a large number of closely related species that populate many different habitats, and HGT may also contribute to this variability. Previous studies, based on a more limited taxonomic sampling, have estimated the core genome of the genus to range approximately between 300 and 2000 protein-coding genes, depending on the number, diversity and quality of the genomes being analysed [8, 9, 12, 28, 72].

Next, we calculated the statistical significance of over/under-representation of each COG functional category within the three core protein sets (100 % hard-core, 95 and 90% soft-cores) of the genus, against the background (File S1, sheet 7). The functional categories of Translation, ribosomal structure and biogenesis (J), Coenzyme transport and metabolism (H), Nucleotide transport and metabolism (F), Energy production and conversion (C), and Post-translational modification, protein turnover and chaperones (O) were consistently over-represented in all three core proteome sets.

### Core proteome of each *Streptomyces* species

For the core/accessory proteome and fingerprint analyses of each individual species, we generated a normalized dataset of five genomes per species (complete genomes had priority over draft genomes, for inclusion). Only 61 species had five or more available genomes each. Furthermore, for only 12 of them were all five genomes of high quality (complete/chromosome assembly). The number of core proteins for each of the 61 species ranged between 4089 and 7132, with an average value of 5542. On average, the core proteome corresponded to 78 % of the proteins of each species, with a minimum of 59 % (C18: *S. melanosporofaciens/violaceusniger/yatensis*) and a maximum of 98 % (C7: *S. rimosus *). The numbers and distribution of the core proteomes of each of the 61 species are shown in Fig. 1, and File S1, sheet 5. For comparison, the various species of *Bacillus* (a genus in the Gram-positive *Firmicutes *) had a core proteome that ranged between 3553 and 5468 proteins (69–91 % of the proteome) (average: 4430; 82 % of the proteome) [43].

The categories of Translation, ribosomal structure and biogenesis (J), Amino acid transport and metabolism (E), Coenzyme transport and metabolism (H), and Carbohydrate transport and metabolism (G) were over-represented in the majority (50-59; >82 %) of the species-level core-proteomes of the 61 species. The category of unknown function (S) was under-represented in the core proteomes of all the species, whereas the Replication, recombination and repair (L) category was under-represented in the core proteomes of 49(80 %) out of 61 species (see File S1, sheet 7).

### Accessory proteome of each *Streptomyces* species

The accessory proteome of each species ranged between 264 and 3225 proteins, with an average value of 1586 (see File S1, sheet 5). For comparison, the accessory proteins per species for the various *Bacillus* species ranged between 192 and 1373 (average: 727) [43]. These observations clearly demonstrate the high inter- and intra-species plasticity of *Streptomycetes* genomes.

### Fingerprint proteins of each species

We define fingerprints as proteins that are present in all members of a given species and at the same time absent in all other species of the genus. Such fingerprints are very important, because they may be involved in species-specific adaptations and may further be used for accurate molecular species typing or as pharmaceutical/biotechnological targets. The total number of fingerprints identified for the 61 species (with at least five genomes each) was 2338, ranging from 0 to 233 per species, with an average number of 38 (see Fig. 1 and File S1, sheet 5). For a complete list of the fingerprint proteins identified in each of the 61 species see File S1, sheet 8. The vast majority of the fingerprints (1753/2338; 75 %) were of unknown function, followed by Transcription (123; 5 %), Amino acid transport and metabolism (87; 4 %), Secondary metabolite biosynthesis, transport and catabolism (77; 3 %), and Carbohydrate transport and metabolism (58; 2.5 %). The hypergeometric test revealed a statistically significant over-representation of the category of unknown function in 53/61 (~87 %) species (for a complete list see File S1, sheet 9). This over-representation of unknown function has also been observed in other fingerprint analyses of *Pseudomonas* [44] and *Bacillus* species [43].

We also examined the number of strict fingerprints of each species. Such strict fingerprints are a subset of the fingerprints that do not have a close homologue (homology threshold of 50 % identity at 50 % of its proteins’ length) in any other *Streptomyces* species. In total, 2281 of the 2338 fingerprints passed this strict threshold.

### Presence and distribution of smBGCs in the various species

The high-quality genomes of 213 *Streptomyces* species were further subjected to antiSMASH analysis (with strict settings). The number of smBGCs identified per species ranged from 16 to 53 (average: 31) (see Figs 1 and 2). A recent study of 205 genomes from 91 species demonstrated low levels of conservation of core enzymes in smBGCs [8]. Many of the smBGCs were strain-specific [8]. Such a high variation may be attributed to HGT in combination with the ecotype [12, 16]. Another significant factor may be the inherently high evolutionary instability of non-ribosomal peptide synthetases (NRPS) and polyketide synthases (PKS), due to gene duplications, HGT, gene loss and shuffling of their modular domain architecture [4, 12, 81, 82].

We further analysed the types of smBGCs and their frequencies in each of the 213 species (see Fig. 2). For this analysis, we only used one high-quality genome per species, with the highest number of smBGCs (for that species). Based on these reference genomes, antiSMASH calculated that 5–23 % (average: 12 %) of the protein-coding genes of each *Streptomyces* species are implicated in secondary metabolism (see File S1, sheet 5). On the other hand, based on COG functional categories, 3–7 % (average 5 %) of the protein-coding genes of each *Streptomyces* species are implicated in secondary metabolism (see File S1, sheet 5). We observed that the ten most frequently present types of smBGCs were encoding genes for the production of terpenes (present in 213/213 species – 100 %), siderophores (213/213 species – 100 %), RiPP-like (other unspecified ribosomally synthesized and post-translationally modified peptide product clusters, present in 200/213 species – 94 %), ectoine (193/213 species – 91 %), non-ribosomal peptide synthetases (NRPS) (191/213 species – 90%), type-I polyketide synthases (PKS) (176/213 species – 83 %), melanin (164/213 species – 77 %), type-III PKS (151/213 species – 71 %), butyrolactone (135/213 species – 63 %), and type-II PKS (126/213 species – 59 %). The complete numbers and types of predicted smBGCs by antiSMASH for each of the high-quality representative genomes (one for each *Streptomyces* species) are available in File S1, sheet 10. Of note, the absolute count of smBGCs may not be sufficient to reveal the full extent of secondary metabolite potential, in terms of the diversity and novelty of their products.

We observed that *Streptomycetes* isolated from terrestrial environments had, on average, more total smBGCs than those isolated from marine environments (31 vs 28; Wilcoxon *P*<0.05; see Fig. S7). We repeated the above analyses (terrestrial vs marine) for each the ten most prevalent categories of smBGCs (see paragraph above) and we observed a statistically significant difference (Wilcoxon *P*<0.05) for terpenes (6.2 vs. 5.5), RiPP-like (2.5 vs. 1.99), melanin (1.21 vs. 0.78) and siderophores (2.71 vs. 2.4) (see Fig. S7). However, in order to control for lineage sampling bias, we gathered one terrestrial and one marine strain (with an available complete genome) from each of 15 different *Streptomyces* species (that had both terrestrial and marine complete genomes available). Next, we performed a paired *t*-test of the number of total smBGCs, the various smBGC categories and the chromosome size of terrestrial versus marine genomes of the same species (File S1, sheet 11). However, we did not observe any statistically significant difference, possibly because of the small sample size (15 pairs). It is also possible that the evolutionary distance between terrestrial and marine strains of the same species is not sufficient to reveal any long-term adaptations in terms of smBGCs. More balanced genomic sampling of marine and terrestrial strains from more *Streptomyces* species will shed further light on this yet unclear interplay between the genome and the environment.

Based on the number of total smBGCs for each of the 213 *Streptomyces* representative genomes, we calculated (for the entire genus) the mean value and the standard deviation. We then identified the actinobacterial genomes that were outliers in terms of numbers of smBGCs (±2 Z-scores) (see Fig. S8). We observed that the majority of actinobacterial genomes (161/192; 84 %) contained significantly fewer smBGCs (−2 Z-scores) compared to the genus *Streptomyces *, while only four of them (2 %) contained significantly more (+2 Z-scores) than in *Streptomycetes*. These four genomes are *Saccharothrix syringae, Kutzneria albida* DSM 43870*, Kibdelosporangium phytohabitans* and *Allokutzneria albata* with the number of smBGCs ranging between 46 and 47. Very similar conclusions were reached when we analysed the percentages of smBGCs (in terms of total chromosomal proteins). This accorded with a previous genome mining study of 102 *Actinomycetes* that revealed that *Streptomyces* was among the genera with very high numbers of smBGCs [6].

### Presence and distribution of CAZymes in different species

The total number of CAZymes per *Streptomyces* species varied significantly, between 73 and 383, with an average value of 191 (see excel File S1, sheet 12, and the respective violin plot in Fig. 1 and heatmap in Fig. 2). A recent study of 205 genomes from 91 species demonstrated low levels of conservation of CAZymes [8].

By far the most abundant of the six CAZyme families was glycoside hydrolase (GH; average: 101 proteins per species), followed by glycosyl-transferase family (GT; average: 34), carbohydrate-binding module family (CBM; average: 32), carbohydrate esterase family (CE; average: 13), polysaccharide lyase family (PL; average: 6) and by a family designated as ‘auxiliary activities’ (AA; average: 6).

We further identified a large monophyletic group within the genus *Streptomyces *, comprising 88 species, that displays a significant expansion of the number of genes encoding these enzymes compared to the other *Streptomycetes* (lineage designated with vertical orange bar in the phylogenomic tree of Fig. 2). More specifically, this lineage has on average 245 enzymes per species, compared to the rest of the genus that has an average of 153 enzymes per species (*t*-test *P*: 1e-23). This difference is mostly attributed to the expansion of the glycoside hydrolase family (130 vs. 80 average number of proteins per species; *t*-test *P*: 3e-22), and the carbohydrate-binding module family (46 vs. 22 average number of proteins per species; *t*-test *P*: 7e-22). A complete list of the CAZymes and their families, identified in each representative genome, is available in File S1, sheet 13.

Next, we investigated whether *Streptomycetes* from certain environments displayed statistically significant differences in the number of CAZymes. We observed that *Streptomyces* strains from terrestrial environments (soil, land plants, land animals) had on average significantly more total CAZymes than *Streptomyces* from marine environments (deep sea, marine plants, marine animals) (197 vs 160; Wilcoxon *P*<0.05) (see Fig. S9). The same holds true for the GH, GT, CBM, CE and PL families of CAZymes. In addition, we observed that *Streptomyces* strains isolated from land plants had significantly more CAZymes than *Streptomyces* strains isolated from land animals (221 vs. 157; Wilcoxon *P*<0.05). The same holds true for the GH, CBM, CE and PL families of CAZymes. In addition, *Streptomyces* strains isolated from marine plants had significantly more CAZymes than *Streptomyces* strains isolated from marine animals (177 vs. 132; Wilcoxon *P*<0.05) (see Fig. S9). In order to control for lineage sampling bias, we repeated the analysis of CAZyme numbers of marine versus terrestrial *Streptomyces* (one strain each from 15 species) as in the above section (of secondary metabolite biosynthetic gene clusters), but did not observe any statistically significant difference. Therefore, a more balanced genomic sampling of marine and terrestrial strains from more *Streptomyces* species is needed in order to shed further light on this yet unclear interplay between the genome and the environment.

We observed that many actinobacterial genomes (89/192; 46 %), contained significantly fewer CAZymes (<−2 Z-scores) than the genus *Streptomyces *, while only seven *Actinobacteria* (4 %) had significantly more CAZymes (>2 Z-scores) than the genus *Streptomyces* (see Fig. S8). More specifically, these seven species were *Saccharothrix syringae, Catenulispora acidiphila* DSM 44928*, Amycolatopsis mediterranei* S699*, Dactylosporangium vinaceum, Nonomuraea gerenzanensis, Actinoplanes derwentensis* and *Couchioplanes caeruleus* that contain 335–486 CAZYmes. Very similar conclusions were also observed when we analysed the CAZymes as percentages of total chromosomal proteins.

Intriguingly, we observed a moderate (*r*>0.4), but statistically significant (*P*<0.05) Pearson correlation between the total number of CAZymes and three categories of smBGCs (siderophores, e-Polylysin and type III lanthipeptides), that are related to competition among bacteria [83–85]. We also observed a weak (*r*=0.24) but statistically significant correlation between the total number of smBGCs and the total number of CAZyme proteins. For this analysis, we used one reference strain from each species, whereas the correlation coefficient *P*-values were corrected using the Benjamini–Hochberg false discovery rate (FDR) method from scipy. The complete correlation matrix that contains each smBGC type and the corresponding correlation values with the total number of CAZymes and each CAZYme family is available in File S1, sheet 14.

### Distribution and conservation of TTA-codon bearing ORFs

We analysed the 213 representative *Streptomyces* genomes for protein-coding ORFs that contain the rare TTA codon that is known to be involved in the regulation of the secondary metabolism and development [33]. All 213 genomes contained between 51 and 505 TTA-bearing ORFs (average: 177). The vast majority (92%) of the TTA-bearing ORFs included only one TTA codon. The total number of TTA-bearing proteins per chromosome varied significantly, even between closely related species (see Fig. 3), thus demonstrating the highly dynamic nature of this type of regulation. The TTA-codon-bearing ORFs were frequently statistically enriched (Hypergeometric test *P*<0.05) for the functional categories of Transcription (in 137/213 species: 64 %), Signal Transduction (in 126/213 species: 59 %), and Secondary Metabolism (in 55/213 species: 26 %), suggesting a hierarchical regulatory cascade (see Fig. 3) [33, 86]. Interestingly, we frequently observed a statistical enrichment for the functional category of Replication/Recombination/Repair (in 119/213 species: 56 %). More specifically, sequences encoding transposases, nucleases and helicases frequently contained TTA codons. It is conceivable that many horizontally transferred genes originated from genera other than *Streptomyces* that are not under the limitations of the rare TTA codon. Such xenologues are expected to either be suppressed within their new genomic environment, perhaps becoming active only under certain physiological conditions, or they might be retained due to the removal of the rare TTA codon. This latter evolutionary scenario accords with our finding that the majority of the TTA codons demonstrate low levels of evolutionary conservation.

Next, we analysed the conservation patterns of these rare codons. We identified 18 orthogroups that had the TTA codon in any position in at least 50 % of the analysed species, whereas one orthogroup had the TTA codon in any position in 87 % (187/213) of the species. We further assessed the conservation of the actual TTA codons in the above-mentioned 18 orthogroups. In total, 11 specific TTA codons from 11 different orthogroups were conserved in at least 50 % of the species that had the corresponding orthogroup. These sites were observed in proteins annotated as DEAD/DEAH box helicase (* S. venezuelae* locus tag: vnz_RS16470 nt pos. 16–19), diguanylate phosphodiesterase (* S. coelicolor* locus tag: FQ762_RS28405 nt pos.1171–1173), aminoglycoside phosphotransferase (* S. coelicolor* locus tag: FQ762_RS37435 nt pos. 328–330), ATP-binding proteins (two different orthogroups; *S. coelicolor* locus tag1: FQ762_RS10100 nt pos. 55–57, *S. coelicolor* locus tag2: FQ762_RS17645 nt pos. 13–15), a hypothetical protein (* S. mirabilis* locus tag: FNV68_RS14920 nt pos. 49–51), helix–turn–helix domain-containing protein (* S. coelicolor* locus tag: FQ762_RS14310 nt pos. 673–675), SpoIIE family protein phosphatase (* S. coelicolor* locus tag: FQ762_RS26065 nt pos. 127–129), an adenylosuccinate lyase (* S. coelicolor* locus tag: FQ762_RS33460 nt pos. 34–36), an alpha/beta hydrolase (* S. coelicolor* locus tag: FQ762_RS22740 nt pos. 163–165) and a DUF742 domain-containing protein (* S. coelicolor* locus tag: FQ762_RS38515 nt pos. 40–42). Reassuringly, the above-mentioned helix–turn–helix domain-containing protein is the AdpA orthologue, whose TTA codon was previously reported to have a functional significance and also be highly conserved [86, 87].

We found that 74 actinobacterial genomes (39 %) contained significantly more TTA-bearing ORFs (>2 Z-scores) than *Streptomyces* (see Fig. S8). Similar conclusions were also observed when we analysed the percentages of TTA-bearing ORFs (as compared to the total chromosomal protein-coding ORFs). Thus, this type of regulation may be relevant in the majority of *Actinobacteria *.

### Intra-species variation of smBGC, CAZyme and TTA-bearing ORF content is high

We analysed the smBGC, CAZyme and TTA-bearing ORF content among strains of the same species, for 12 species with five high-quality genomes each (denoted with red stars in the phylogenomic tree of Fig. 2). We only used complete genomes for this analysis, to ensure that smBGCs were intact. The intra-species variation in terms of content was estimated by the Jaccard distance (see File S1, sheet 15). We observed that the variation of smBGC, CAZyme and TTA-bearing ORF content among strains of the same species could be quite high. Some species are homogenous, while others may vary greatly (see violin plots of Fig. 1). Therefore, genomic mining for such biotechnologically important enzymes and regulatory proteins is preferable at the strain level, rather than the species level, whenever possible.

### *Streptomyces* genus-level core protein-coding genes tend to be found in the central region of the chromosome

Previous analyses have shown that the core genes of the genus *Streptomyces* are frequently situated at the central portion of the linear chromosome [9, 88]. To further assess this previous observation, but this time with a significantly higher number of high-quality genomes, we split the linear chromosome into three parts, the two arms (25 % of the total length each) and the centre in-between (50 % of the total length), and performed the hypergeometric test on the number of genus-level core protein-coding genes present in each part. Over/under-representation of these core genes was based on a *P*-value cut-off of 0.05 and ≥5 % change from the background frequency. At the genus level, the 100 % hard-core, and the 95 and 90% soft-core protein-coding genes are over-represented at the centre of the chromosome in 196, 192 and 190 (out of 213) species, respectively (see File S1, sheet 16).

### *Streptomyces* species-level core protein-coding genes have a weaker tendency to be found in the central region of the chromosome

For the 61 species for which we identified their species-level core proteomes, we observed that (i) in 31 (51 %) of them, genes for their species-level core proteins did not demonstrate any statistically significant over-representation in the central region, (ii) 28 (46 %) of them had a statistically significant over-representation of core genes in the central region of the chromosome, and (iii) two (3 %) of them had a statistically significant under-representation (see File S1, sheet 16).

### Fingerprints, smBGCs, CAZymes and TTA-codon bearing ORFs tend to localize at the arms of the chromosome

We investigated the location of fingerprint genes, smBGCs, CAZymes and TTA-bearing ORFs within the chromosomes of the 213 *Streptomyces* species for which high-quality genomes were available. We observed that smBGCs were statistically over-represented on the arms of the chromosome in 161 (76 %) species, whereas the CAZymes were also statistically over-represented on the arms of the chromosome in 104 (49 %) species. We repeated the same localization analyses as above for the 26 *Streptomyces* species with more than 30 fingerprints each and observed that they were mostly over-represented on the chromosome arms (in 18/26 genomes; 69 %) (see File S1, sheet 16). We also observed that the TTA-bearing ORFs were over-represented (hypergeometric test, *P*<0.05) on the chromosome arms in 105/213 species, whereas they were under-represented on the chromosome arms in 18/213 species.

Previous studies of *Streptomycetes* and other *Actinobacteria* (based on fewer genomes) have demonstrated the evolutionary stability of the central region of the chromosome and the elevated volatility of the chromosome arms, where many insertions/deletions, chromosomal re-arrangements and HGT integration events frequently occur [5, 9, 10, 88–92]. In addition, the chromosome centre displays synteny among various members of the *Actinomycetales* [91]. Thus, the chromosome arms clearly emerge as the part of the genome that is mainly responsible for rapid evolutionary adaptation at both the species and strain level.

## Conclusions

This is, to date, the most comprehensive large-scale comparative analysis of the genomic plasticity of the *Streptomycetes* at the genus and species level. It is based on 742 genomes from 213 *Streptomyces* species, and goes beyond a recent study by Otani *et al*. [8] (based on 205 genomes from 91 species) by applying a more refined analysis pipeline that stratifies orthologues/homologues at different levels of stringency, identifies any unique proteins characterizing different species as species-specific fingerprints, and considers the bldA-dependent TTA-containing genes known to be important for developmental regulation of metabolism and cell morphology. In addition, it puts many of these results within the wider context of the phylum *Actinobacteria* (192 genera). Our analysis demonstrates very high variability among the 213 species of the genus, in terms of (i) ecology, (ii) total chromosomal protein-coding genes, (iii) species-level core proteins, (iv) species-level accessory proteins, (v) species-level fingerprint proteins, (vi) CAZymes, (vii) smBGCs and (viii) ORFs bearing the rare TTA codons, even among strains of the same species, thus highlighting the need for strain-specific genome mining for these biotechnologically important enzymes.

*Streptomycetes* have significantly more smBGCs and CAZymes than most of the other *Actinobacteria *. Intriguingly, the total number of CAZymes moderately correlates (*r*>0.4) with three categories of smBGCs (siderophores, e-Polylysin and type III lanthipeptides) that are related to competition among bacteria. Therefore, *Streptomyces* species not only have a high number of biopolymer-degrading enzymes to obtain nutrients, but also evolve an arsenal of siderophores and antimicrobial agents to suppress competition by other bacteria in the nutrient-rich environments that they create.

We observed that the genus-level core proteins tend to be located at the central region of the chromosome, whereas the evolutionarily volatile species-level fingerprints, genes for smBGCs, CAZymes and TTA-codon-bearing ORFs tend to be located at the arms of the chromosome. Thus, the arms of the chromosome (the regions that are distal to the replication origin at the centre [93]) clearly emerge as the part of the genome that is mainly responsible for rapid adaptation at both the species and strain level. The genus *Streptomyces* has undergone significant genomic expansion. Importantly, the extent of paralogy in the *Streptomycetes* genomes is significantly higher than that observed in many other (though not all) *Actinobacteria *, and in *Bacillus *. A few notable cases of high proportions of very recent gene duplicates are mainly due to block duplications at the chromosome arms.

The high proportion of regulatory protein-coding gene content of the *Streptomyces* species, the high volatility of the species-level core/accessory proteins, smBGCs, CAZymes, TTA-bearing ORFs, the high proportion of paralogues, the already complex genome of the *Streptomyces* LCA as well as the evolutionary volatility of the chromosome arms may all explain the remarkably rapid adaptability of this genus to very diverse environments. All the above also strongly suggest that the various *Streptomycetes* genomes may be amenable to extensive genome reduction for the construction of minimal genomes with industrial applications. Within a controlled artificial environment, a minimal genome will not need many of the genomic components that are highly volatile and necessary for multicellular development and rapid adaptation to certain natural and complex environments.

## Supplementary Data

Supplementary material 1

Supplementary material 2

Supplementary material 3

## References

[1] Nikolaidis M, Hesketh A, Frangou N, Mossialos D, Van de Peer Y (2023). FigShare.

[2] Traxler MF, Rozen DE (2022). Ecological drivers of division of labour in *Streptomyces*. Curr Opin Microbiol.

[3] Chater KF, Biró S, Lee KJ, Palmer T, Schrempf H (2010). The complex extracellular biology of *Streptomyces*. FEMS Microbiol Rev.

[4] Seshadri R, Roux S, Huber KJ, Wu D, Yu S (2022). Expanding the genomic encyclopedia of *Actinobacteria* with 824 isolate reference genomes. Cell Genom.

[5] Zhou Z, Gu J, Li Y-Q, Wang Y (2012). Genome plasticity and systems evolution in *Streptomyces*. BMC Bioinformatics.

[6] Doroghazi JR, Metcalf WW (2013). Comparative genomics of actinomycetes with a focus on natural product biosynthetic genes. BMC Genomics.

[7] Antimicrobial Resistance Collaborators (2022). Global burden of bacterial antimicrobial resistance in 2019: a systematic analysis. Lancet.

[8] Otani H, Udwary DW, Mouncey NJ (2022). Comparative and pangenomic analysis of the genus *Streptomyces*. Sci Rep.

[9] Lorenzi J-N, Lespinet O, Leblond P, Thibessard A (2019). Subtelomeres are fast-evolving regions of the *Streptomyces* linear chromosome. Microb Genom.

[10] Tidjani A-R, Lorenzi J-N, Toussaint M, van Dijk E, Naquin D (2019). Massive gene flux drives genome diversity between sympatric *Streptomyces* conspecifics. mBio.

[11] Land M, Hauser L, Jun S-R, Nookaew I, Leuze MR (2015). Insights from 20 years of bacterial genome sequencing. Funct Integr Genomics.

[12] McDonald BR, Currie CR, Keim P, Marx C, Abbot P (2017). Lateral gene transfer dynamics in the ancient bacterial genus *Streptomyces*. mBio.

[13] Bentley SD, Chater KF, Cerdeño-Tárraga A-M, Challis GL, Thomson NR (2002). Complete genome sequence of the model actinomycete *Streptomyces coelicolor* A3(2). Nature.

[14] Andam CP, Choudoir MJ, Vinh Nguyen A, Sol Park H, Buckley DH (2016). Contributions of ancestral inter-species recombination to the genetic diversity of extant *Streptomyces* lineages. ISME J.

[15] Doroghazi JR, Buckley DH (2010). Widespread homologous recombination within and between *Streptomyces* species. ISME J.

[16] Xu L, Ye K-X, Dai W-H, Sun C, Xu L-H (2019). Comparative genomic insights into secondary metabolism biosynthetic gene cluster distributions of marine *Streptomyces*. Mar Drugs.

[17] Salam N, Jiao J-Y, Zhang X-T, Li W-J (2020). Update on the classification of higher ranks in the phylum *Actinobacteria*. Int J Syst Evol Microbiol.

[18] Chater KF, Chandra G (2006). The evolution of development in *Streptomyces* analysed by genome comparisons. FEMS Microbiol Rev.

[19] Labeda DP, Goodfellow M, Brown R, Ward AC, Lanoot B (2012). Phylogenetic study of the species within the family *Streptomycetaceae*. Antonie van Leeuwenhoek.

[20] Han J-H, Cho M-H, Kim SB (2012). Ribosomal and protein coding gene based multigene phylogeny on the family *Streptomycetaceae*. Syst Appl Microbiol.

[21] Labeda DP, Dunlap CA, Rong X, Huang Y, Doroghazi JR (2017). Phylogenetic relationships in the family Streptomycetaceae using multi-locus sequence analysis. Antonie van Leeuwenhoek.

[22] Alam MT, Merlo ME, Takano E, Breitling R (2010). Genome-based phylogenetic analysis of *Streptomyces* and its relatives. Mol Phylogenet Evol.

[23] Nouioui I, Carro L, García-López M, Meier-Kolthoff JP, Woyke T (2018). Genome-based taxonomic classification of the phylum *Actinobacteria*. Front Microbiol.

[24] Gomez-Escribano JP, Alt S, Bibb MJ (2016). Next generation sequencing of *Actinobacteria* for the discovery of novel natural products. Mar Drugs.

[25] Jackson SA, Crossman L, Almeida EL, Margassery LM, Kennedy J (2018). Diverse and abundant secondary metabolism biosynthetic gene clusters in the genomes of marine sponge derived *Streptomyces* spp. isolates. Mar Drugs.

[26] Belknap KC, Park CJ, Barth BM, Andam CP (2020). Genome mining of biosynthetic and chemotherapeutic gene clusters in *Streptomyces* bacteria. Sci Rep.

[27] Lee N, Kim W, Hwang S, Lee Y, Cho S (2020). Thirty complete Streptomyces genome sequences for mining novel secondary metabolite biosynthetic gene clusters. Sci Data.

[28] Caicedo-Montoya C, Manzo-Ruiz M, Ríos-Estepa R (2021). Pan-genome of the genus *Streptomyces* and prioritization of biosynthetic gene clusters with potential to produce antibiotic compounds. Front Microbiol.

[29] Seipke RF (2015). Strain-level diversity of secondary metabolism in *Streptomyces albus*. PLoS One.

[30] Vicente CM, Thibessard A, Lorenzi J-N, Benhadj M, Hôtel L (2018). Comparative genomics among closely related *Streptomyces* strains revealed specialized metabolite biosynthetic gene cluster diversity. Antibiotics.

[31] Park CJ, Andam CP (2019). Within-species genomic variation and variable patterns of recombination in the tetracycline producer *Streptomyces rimosus*. Front Microbiol.

[32] Martinet L, Naômé A, Baiwir D, De Pauw E, Mazzucchelli G (2020). On the risks of phylogeny-based strain prioritization for drug discovery: *Streptomyces lunaelactis* as a case study. Biomolecules.

[33] Chater KF, Chandra G (2008). The use of the rare UUA codon to define “expression space” for genes involved in secondary metabolism, development and environmental adaptation in *Streptomyces*. J Microbiol.

[34] Kim D-W, Chater KF, Lee K-J, Hesketh A (2005). Effects of growth phase and the developmentally significant bldA-specified tRNA on the membrane-associated proteome of Streptomyces coelicolor. Microbiology.

[35] Hesketh A, Bucca G, Laing E, Flett F, Hotchkiss G (2007). New pleiotropic effects of eliminating a rare tRNA from *Streptomyces coelicolor*, revealed by combined proteomic and transcriptomic analysis of liquid cultures. BMC Genomics.

[36] Bu Q-T, Li Y-P, Xie H, Wang J, Li Z-Y (2020). Comprehensive dissection of dispensable genomic regions in *Streptomyces* based on comparative analysis approach. Microb Cell Fact.

[37] McDaniel R, Ebert-Khosla S, Hopwood DA, Khosla C (1993). Engineered biosynthesis of novel polyketides. Science.

[38] Gomez-Escribano JP, Bibb MJ (2011). Engineering *Streptomyces coelicolor* for heterologous expression of secondary metabolite gene clusters. Microb Biotechnol.

[39] Komatsu M, Uchiyama T, Ōmura S, Cane DE, Ikeda H (2010). Genome-minimized *Streptomyces* host for the heterologous expression of secondary metabolism. Proc Natl Acad Sci.

[40] Myronovskyi M, Rosenkränzer B, Nadmid S, Pujic P, Normand P (2018). Generation of a cluster-free *Streptomyces albus* chassis strains for improved heterologous expression of secondary metabolite clusters. Metab Eng.

[41] Ahmed Y, Rebets Y, Estévez MR, Zapp J, Myronovskyi M (2020). Engineering of *Streptomyces lividans* for heterologous expression of secondary metabolite gene clusters. Microb Cell Fact.

[42] Huguet-Tapia JC, Lefebure T, Badger JH, Guan D, Pettis GS (2016). Genome content and phylogenomics reveal both ancestral and lateral evolutionary pathways in plant-pathogenic *Streptomyces* species. Appl Environ Microbiol.

[43] Nikolaidis M, Hesketh A, Mossialos D, Iliopoulos I, Oliver SG (2022). A comparative analysis of the core proteomes within and among the *Bacillus subtilis* and *Bacillus cereus* evolutionary groups reveals the patterns of lineage- and species-specific adaptations. Microorganisms.

[44] Nikolaidis M, Mossialos D, Oliver SG, Amoutzias GD (2020). Comparative analysis of the core proteomes among the *Pseudomonas* major evolutionary groups reveals species-specific adaptations for *Pseudomonas aeruginosa* and *Pseudomonas chlororaphis*. Diversity.

[45] Emms DM, Kelly S (2019). OrthoFinder: phylogenetic orthology inference for comparative genomics. Genome Biol.

[46] Edgar RC (2004). MUSCLE: multiple sequence alignment with high accuracy and high throughput. Nucleic Acids Research.

[47] Castresana J (2000). Selection of conserved blocks from multiple alignments for their use in phylogenetic analysis. Mol Biol Evol.

[48] Minh BQ, Schmidt HA, Chernomor O, Schrempf D, Woodhams MD (2020). Corrigendum to: IQ-TREE 2: new models and efficient methods for phylogenetic inference in the genomic era. Mol Biol Evol.

[49] Chevenet F, Brun C, Bañuls A-L, Jacq B, Christen R (2006). TreeDyn: towards dynamic graphics and annotations for analyses of trees. BMC Bioinformatics.

[50] Letunic I, Bork P (2021). Interactive Tree Of Life (iTOL) v5: an online tool for phylogenetic tree display and annotation. Nucleic Acids Res.

[51] Anisimova M, Gascuel O (2006). Approximate likelihood-ratio test for branches: a fast, accurate, and powerful alternative. Syst Biol.

[52] Jain C, Rodriguez-R LM, Phillippy AM, Konstantinidis KT, Aluru S (2018). High throughput ANI analysis of 90K prokaryotic genomes reveals clear species boundaries. Nat Commun.

[53] Enright AJ, Van Dongen S, Ouzounis CA (2002). An efficient algorithm for large-scale detection of protein families. Nucleic Acids Res.

[54] Vlastaridis P, Kyriakidou P, Chaliotis A, Van de Peer Y, Oliver SG (2017). Estimating the total number of phosphoproteins and phosphorylation sites in eukaryotic proteomes. GigaScience.

[55] Cantalapiedra CP, Hernández-Plaza A, Letunic I, Bork P, Huerta-Cepas J (2021). eggNOG-mapper v2: functional annotation, orthology assignments, and domain prediction at the metagenomic scale. Mol Biol Evol.

[56] Huerta-Cepas J, Szklarczyk D, Heller D, Hernández-Plaza A, Forslund SK (2019). eggNOG 5.0: a hierarchical, functionally and phylogenetically annotated orthology resource based on 5090 organisms and 2502 viruses. Nucleic Acids Res.

[57] Kanehisa M, Furumichi M, Tanabe M, Sato Y, Morishima K (2017). KEGG: new perspectives on genomes, pathways, diseases and drugs. Nucleic Acids Res.

[58] Tatusov RL, Galperin MY, Natale DA, Koonin EV. (2000). The COG database: a tool for genome-scale analysis of protein functions and evolution. Nucleic Acids Res.

[59] Virtanen P, Gommers R, Oliphant TE, Haberland M, Reddy T (2020). Author correction: SciPy 1.0: fundamental algorithms for scientific computing in python. Nat Methods.

[60] Blin K, Shaw S, Kloosterman AM, Charlop-Powers Z, van Wezel GP (2021). antiSMASH 6.0: improving cluster detection and comparison capabilities. Nucleic Acids Res.

[61] Zhang H, Yohe T, Huang L, Entwistle S, Wu P (2018). dbCAN2: a meta server for automated carbohydrate-active enzyme annotation. Nucleic Acids Res.

[62] Drula E, Garron M-L, Dogan S, Lombard V, Henrissat B (2022). The carbohydrate-active enzyme database: functions and literature. Nucleic Acids Res.

[63] Cabanettes F, Klopp C (2018). D-GENIES: dot plot large genomes in an interactive, efficient and simple way. PeerJ.

[64] Li H, Alkan C (2021). New strategies to improve minimap2 alignment accuracy. Bioinformatics.

[65] Altenhoff AM, Levy J, Zarowiecki M, Tomiczek B, Warwick Vesztrocy A (2019). OMA standalone: orthology inference among public and custom genomes and transcriptomes. Genome Res.

[66] Train C-M, Pignatelli M, Altenhoff A, Dessimoz C, Schwartz R (2019). iHam and pyHam: visualizing and processing hierarchical orthologous groups. Bioinformatics.

[67] Vela Gurovic MS, Díaz ML, Gallo CA, Dietrich J (2021). Phylogenomics, CAZyome and core secondary metabolome of *Streptomyces albus* species. Mol Genet Genomics.

[68] Komaki H, Ichikawa N, Oguchi A, Hamada M, Tamura T (2017). Genome analysis-based reclassification of *Streptomyces endus* and *Streptomyces sporocinereus* as later heterotypic synonyms of *Streptomyces hygroscopicus* subsp. hygroscopicus. Int J Syst Evol Microbiol.

[69] Komaki H, Tamura T (2019). Reclassification of *Streptomyces rimosus* subsp. paromomycinus as *Streptomyces paromomycinus* sp. nov. Int J Syst Evol Microbiol.

[70] Madhaiyan M, Saravanan VS, See-Too W-S (2020). Genome-based analyses reveal the presence of 12 heterotypic synonyms in the genus *Streptomyces* and emended descriptions of *Streptomyces bottropensis*, *Streptomyces celluloﬂavus*, *Streptomyces fulvissimus*, *Streptomyces glaucescens*, *Streptomyces murinus*, and *Streptomyces variegatus*. Int J Syst Evol Microbiol.

[71] Lee N, Choi M, Kim W, Hwang S, Lee Y (2021). Re-classification of *Streptomyces venezuelae* strains and mining secondary metabolite biosynthetic gene clusters. iScience.

[72] Kim J-N, Kim Y, Jeong Y, Roe J-H, Kim B-G (2015). Comparative genomics reveals the core and accessory genomes of *Streptomyces* species. J Microbiol Biotechnol.

[73] Ranea JAG, Grant A, Thornton JM, Orengo CA (2005). Microeconomic principles explain an optimal genome size in bacteria. Trends Genet.

[74] Levine M, Tjian R (2003). Transcription regulation and animal diversity. Nature.

[75] van Nimwegen E (2003). Scaling laws in the functional content of genomes. Trends Genet.

[76] Freeling M, Thomas BC (2006). Gene-balanced duplications, like tetraploidy, provide predictable drive to increase morphological complexity. Genome Res.

[77] Maere S, De Bodt S, Raes J, Casneuf T, Van Montagu M (2005). Modeling gene and genome duplications in eukaryotes. Proc Natl Acad Sci.

[78] Gevers D, Vandepoele K, Simillon C, Van de Peer Y (2004). Gene duplication and biased functional retention of paralogs in bacterial genomes. Trends Microbiol.

[79] Gogarten JP, Townsend JP (2005). Horizontal gene transfer, genome innovation and evolution. Nat Rev Microbiol.

[80] Kunin V, Ouzounis CA (2003). The balance of driving forces during genome evolution in prokaryotes. Genome Res.

[81] Amoutzias GD, Van de Peer Y, Mossialos D (2008). Evolution and taxonomic distribution of nonribosomal peptide and polyketide synthases. Fute Microb.

[82] Amoutzias GD, Chaliotis A, Mossialos D (2016). Discovery strategies of bioactive compounds synthesized by nonribosomal peptide synthetases and type-I polyketide synthases derived from marine microbiomes. Mar Drugs.

[83] Kramer J, Özkaya Ö, Kümmerli R (2020). Bacterial siderophores in community and host interactions. Nat Rev Microbiol.

[84] Wang L, Zhang C, Zhang J, Rao Z, Xu X (2021). Epsilon-poly-L-lysine: recent advances in biomanufacturing and applications. Front Bioeng Biotechnol.

[85] Gomes KM, Duarte RS, de Freire Bastos M do C (2017). Lantibiotics produced by Actinobacteria and their potential applications (a review). Microbiology.

[86] Chandra G, Chater KF (2014). Developmental biology of *Streptomyces* from the perspective of 100 actinobacterial genome sequences. FEMS Microbiol Rev.

[87] Rabyk M, Yushchuk O, Rokytskyy I, Anisimova M, Ostash B (2018). Genomic insights into evolution of AdpA family master regulators of morphological differentiation and secondary metabolism in *Streptomyces*. J Mol Evol.

[88] Choulet F, Aigle B, Gallois A, Mangenot S, Gerbaud C (2006). Evolution of the terminal regions of the *Streptomyces* linear chromosome. Mol Biol Evol.

[89] Volff JN, Altenbuchner J (1998). Genetic instability of the *Streptomyces* chromosome. Mol Microbiol.

[90] Chen CW, Huang C-H, Lee H-H, Tsai H-H, Kirby R (2002). Once the circle has been broken: dynamics and evolution of *Streptomyces* chromosomes. Trends Genet.

[91] Kirby R (2011). Chromosome diversity and similarity within the *Actinomycetales*. FEMS Microbiol Lett.

[92] Hoff G, Bertrand C, Piotrowski E, Thibessard A, Leblond P (2018). Genome plasticity is governed by double strand break DNA repair in *Streptomyces*. Sci Rep.

[93] Musialowski MS, Flett F, Scott GB, Hobbs G, Smith CP (1994). Functional evidence that the principal DNA replication origin of the *Streptomyces coelicolor* chromosome is close to the dnaA-gyrB region. J Bacteriol.

