# Supplementary material for: A panoramic view of the genomic landscape of the genus Streptomyces
Source: Microb Genom. 2023 Jun 2;9(6):mgen001028. doi: 10.1099/mgen.0.001028 (PMC10327506; doi:10.1099/mgen.0.001028)

Supplementary figure 1: *Streptomyces* phylogenomic tree

The phylogenomic tree is based on 742 genomes (355 high quality, 387 draft) and was calculated from 104 core proteins (17,779 sites) using the QInsect+F+I+G model in the IQ-Tree2 software. On the first column next to the tree we show the genome NCBI annotation. On the second column is the FastANI species cluster. On the third column with red dots we denote the complete genomes. On the fourth column is the number of chromosomal proteins. On the fifth column is the habitat annotation from NCBI. On the sixth column is the host annotation from NCBI.

●Species annotations that are found in more than one species clusters (based on FastANI) are hightlighted in light red and can be seen in Table1.

●The species clusters that contain more than one species names are shown inside red boxes. The 3 non-monophyletic species clusters that also contain multiple species names are shown inside dotted red boxes.

●The 54 *Streptomyces* complete genomes isolated from marine environments are highlighted in blue.

Table1: Species names that are found in multiple species clusters

Species name	FastANI species clusters
S. venezuelae	C10;C126;C128;C131;C154;C167;C179
S. albus	C0;C24;C156;C207
S. griseus	C149;C156;C158;C181
S. lavendulae	C47;C121;C169
S. fradiae	C16;C25;C198
S. lydicus	C17;C26;C193
S. collinus	C19;C115
S. incarnatus	C91;C115
S. seoulensis	C119;C124
S. formicae	C129;C145
S. niveus	C165;C182
S. virginiae	C44;C169
S. tsukubensis	C54;C186
S. mobaraensis	C56;C201
S. hygroscopicus	C22;C49
S. microflavus	C23;C45
S. albulus	C27;C52
S. chartreusis	C31;C32
S. antibioticus	C34;C67

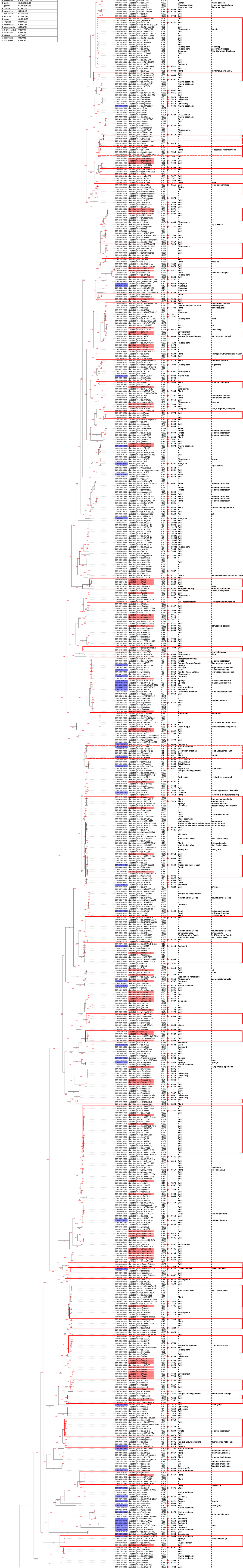

Supplementary figure 2: *Streptomyces* COG categories comparison

COG functional categories (% of proteome) comparison of the 213 members (one from each species) of the *Streptomyces* genus against that of i) 192 representatives (one from each genus) of the Actinobacteria phylum and ii) 55 representatives (one from each species) of the *Bacillus* genus

●The COG categories were compared using the t-test for samples with unequal variance and a p-value cut-off of 0.05. P-values have been corrected using the Benjamini-Hochberg FDR method. Statistically significant results are marked with an asterisk (*)

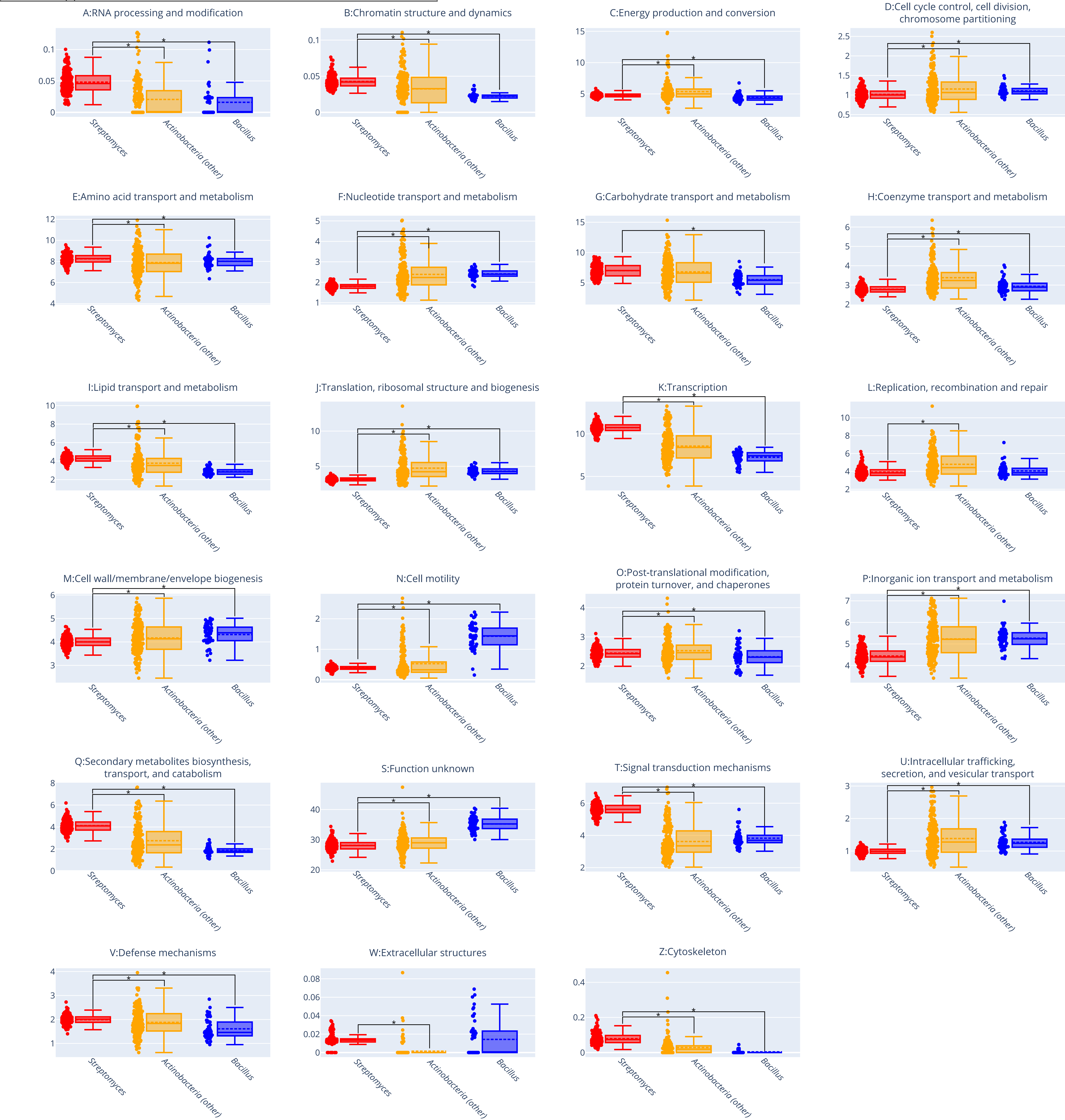

Supplementary figure 3: COG categories of Actinobacteria

A) Phylogenomic tree of 192 Actinobacterial genera (one representative from each genus, whenever available). The horizontal bars next to the tree correspond to the total number of chromosomal protein-coding loci per representative genome (light blue), the absolute number of proteins (for that genome) that belong to the functional categories of Transcription (red), Signal Transduction (yellow), Lipid Transport and metabolism (green), Secondary Metabolism (blue). B) Boxplots of the percentages (per proteome) of the proteins that belong to one of the four above mentioned functional categories, for *Streptomyces* (213 species), Actinobacteria (192 genera) and *Bacillus* (55 species).

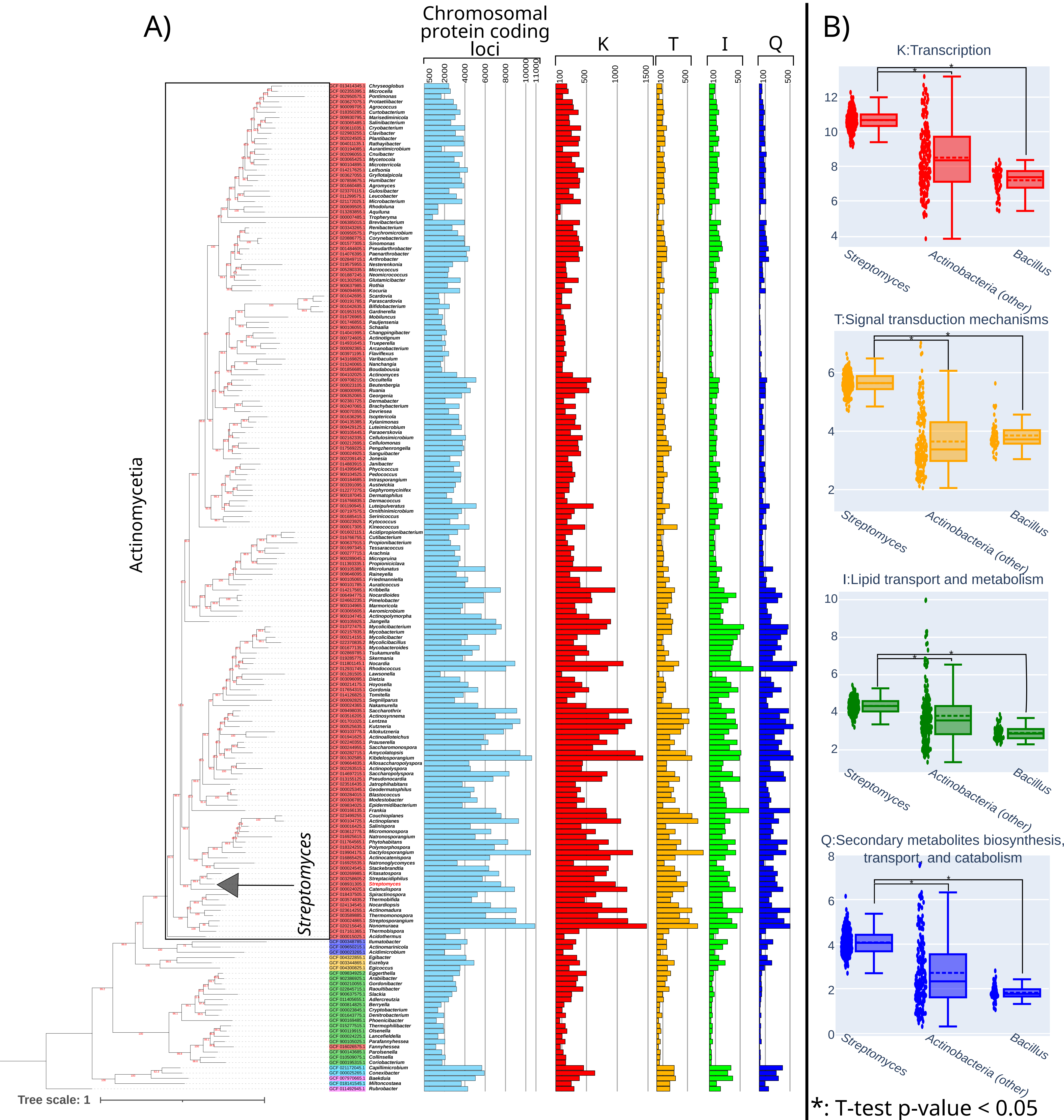

Supplementary figure 4: Percentage of paralogues in different cutoffs

Percentages of chromosomal proteins being singletons and homologs (five amino acid identity bins: 51-60%, 61-70%, 71-80%, 81-90%, 91-100%) in the 213 species. Transposases have been removed from this analysis. B) Boxplot of the percentage of singletons and homologs for each of the five amino acid identity bins. C) Boxplots of the percentage of chromosomal proteins being singletons and homologs (five amino acid identity bins/ cutoffs) in the genomes of 3 different taxonomic groups. The 213 *Streptomyces* genomes were compared to 192 Actinobacterial and 55 *Bacillus*.

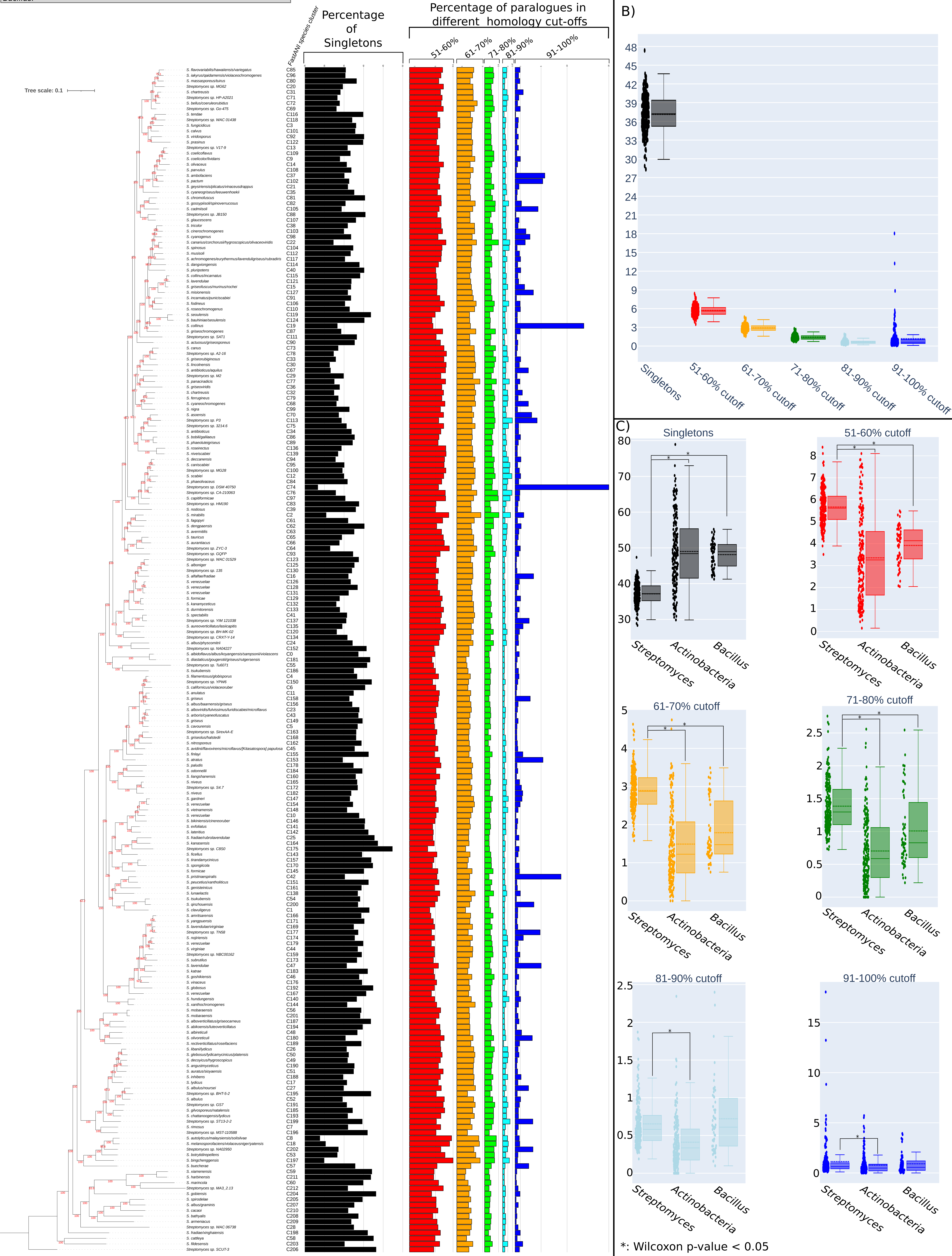

Supplementary figure 5: Recent gene duplications in eight *Streptomyces* genomes

In order to investigate very recent and extensive lineage-specific expansions of certain *Streptomyces* species, genome-scale dotplots of each of the genomes against itself were performed with the D-genies software (default parameters) and the Minimap2 v2.24 aligner.

Below each genome dotplot we show the genome diagrams with the recent paralogues. Recent paralogues were calculated using the 91-100% aminoacid identity cut-off and are shown in red color. Vertical bars above the diagram are genes located on the positive strand, while vertical bars below are genes located on the negative strand. The diagrams were created using the Biopython library.

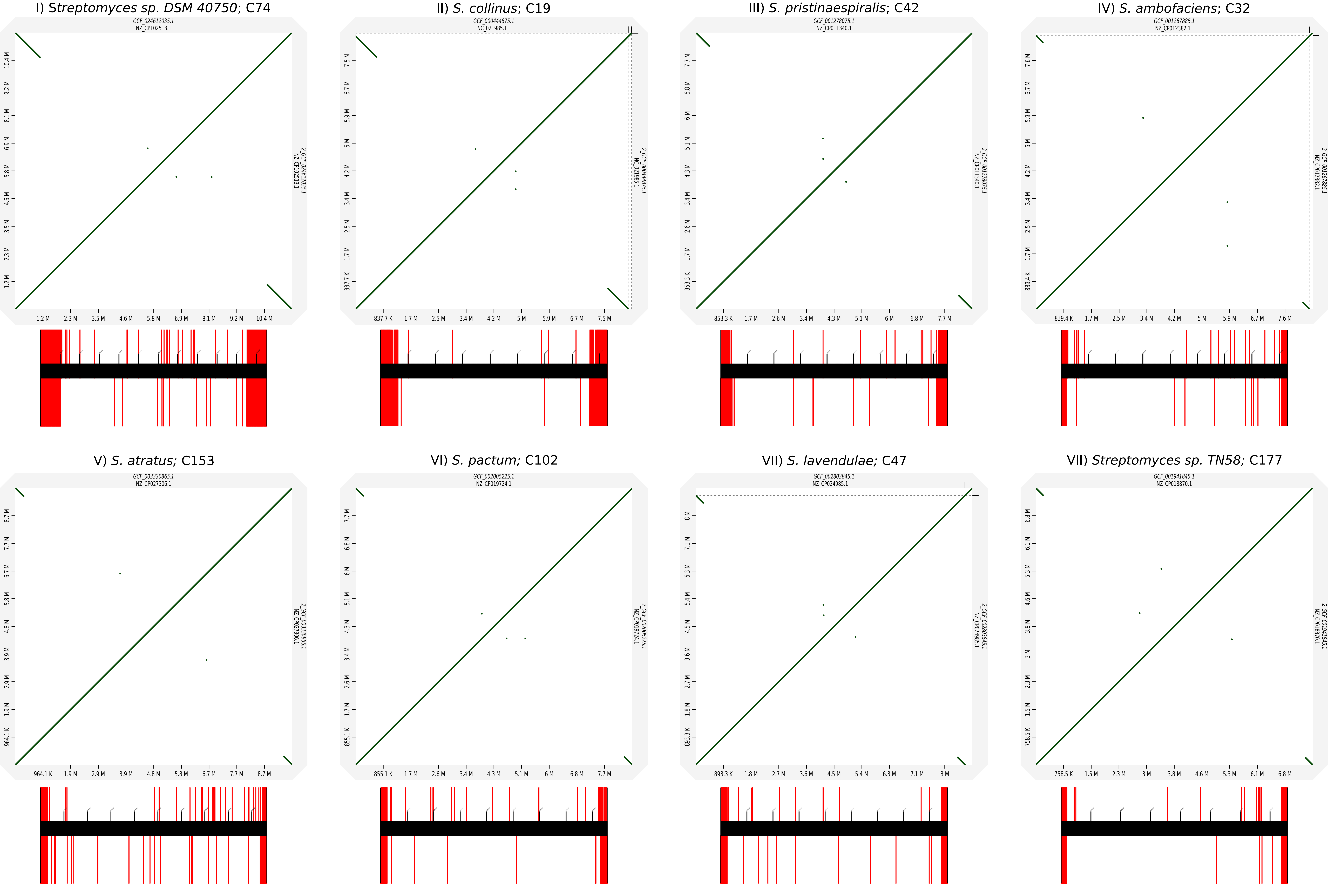

Supplementary figure 6: *Streptomycetaceae* HOGs

HOGs were calculated using the OMA standalone software v.2.5 with the pre-computed phylogenomic tree as input. The rest of the parameters were set to default.

Gene evolution profiling and visualization was achieved with pyHAM.

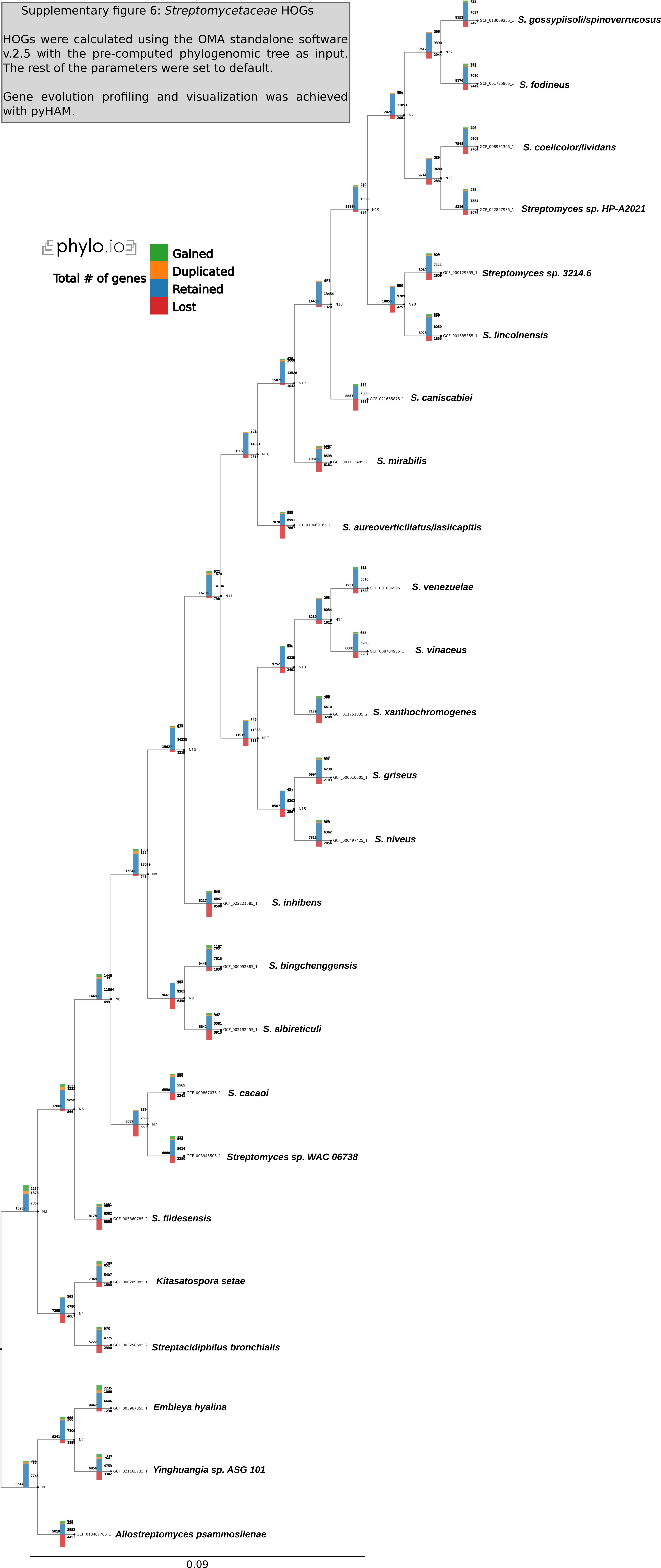

Supplementary figure 7: smBGC comparison in different habitats

The habitat annotation for each of the 355 *Streptomyces* genomes was downloaded from Genbank. Only genomes with habitat annotation were used in this analysis. The 10 most commonly observed smBGC products are shown.

Visualization was achieved using the plotly graphics library.

Statistical significance was calculated using the Wilcoxon test (p-value < 0.05). P-values have been adjusted using multiple testing correction with FDR method

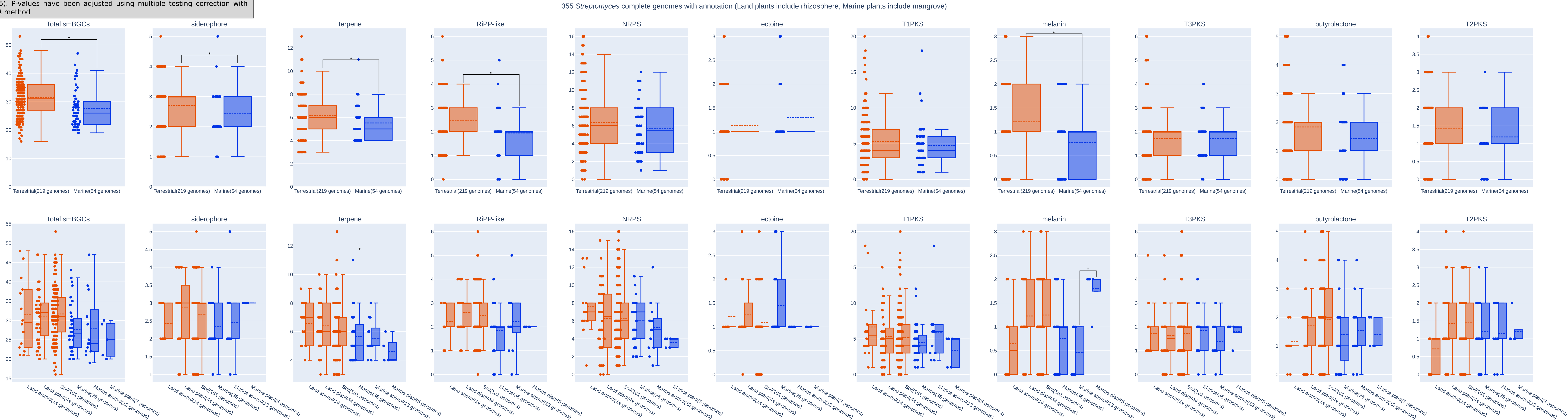

Supplementary figure 8: Comparison of percentages of smBGCs, CAZYmes and TTA-bearing proteins between *Streptomyces* and Actinobacteria

The boxplots on the left of the phylogenomic tree represent the percentage of smBGCs, CAZYmes and TTA-bearing proteins in *Streptomyces* (213 genomes) and Actinobacteria (192 genomes). Statistical significance was calculated using the Wilcoxon test (p-value < 0.05).

The horizontal bars next to the tree represent the number of chromosomally encoded proteins, the percentage of smBGCs, the percentage of CAZYmes and the percentage of TTA-bearing proteins in the Actinobacterial genomes.

The red vertical dotted bars indicate the *Streptomyces* -+ 2Z-scores interval for that specific feature.

The black vertical dotted bar indicates the value of *Streptomyces coelicolor* reference genome for that specific feature.

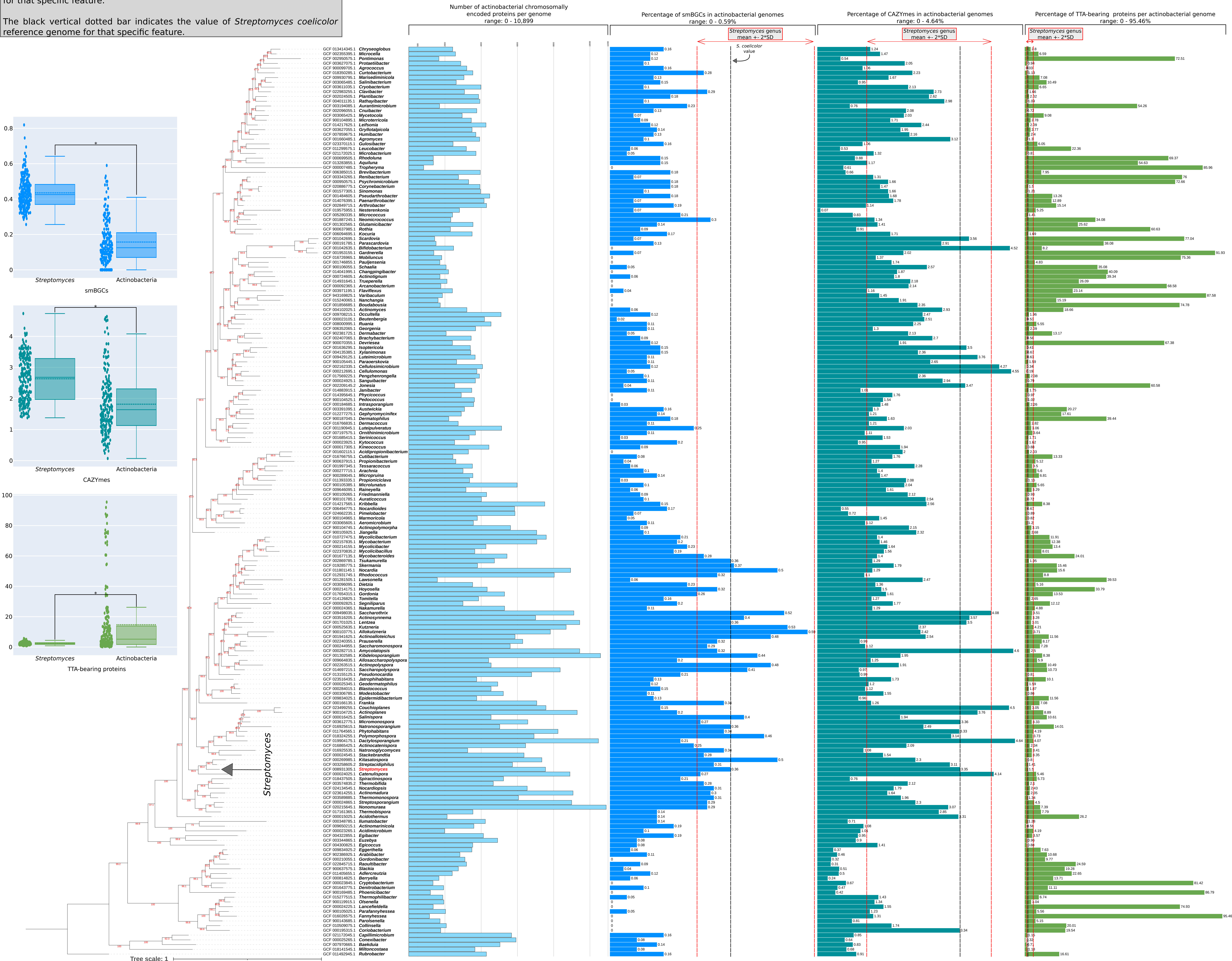

Supplementary figure 9: CAZYmes comparison in different habitats

The habitat annotation for each of the 355 *Streptomyces* genomes was downloaded from Genbank. Only genomes with habitat annotation were used in this analysis.

Visualization was achieved using the plotly graphics library.

Statistical significance was calculated using the Wilcoxon test (p-value < 0.05). P-values have been adjusted using multiple testing correction with FDR method

355 *Streptomyces* complete genomes with annotation (Land plants include rhizosphere, Marine plants include mangrove)

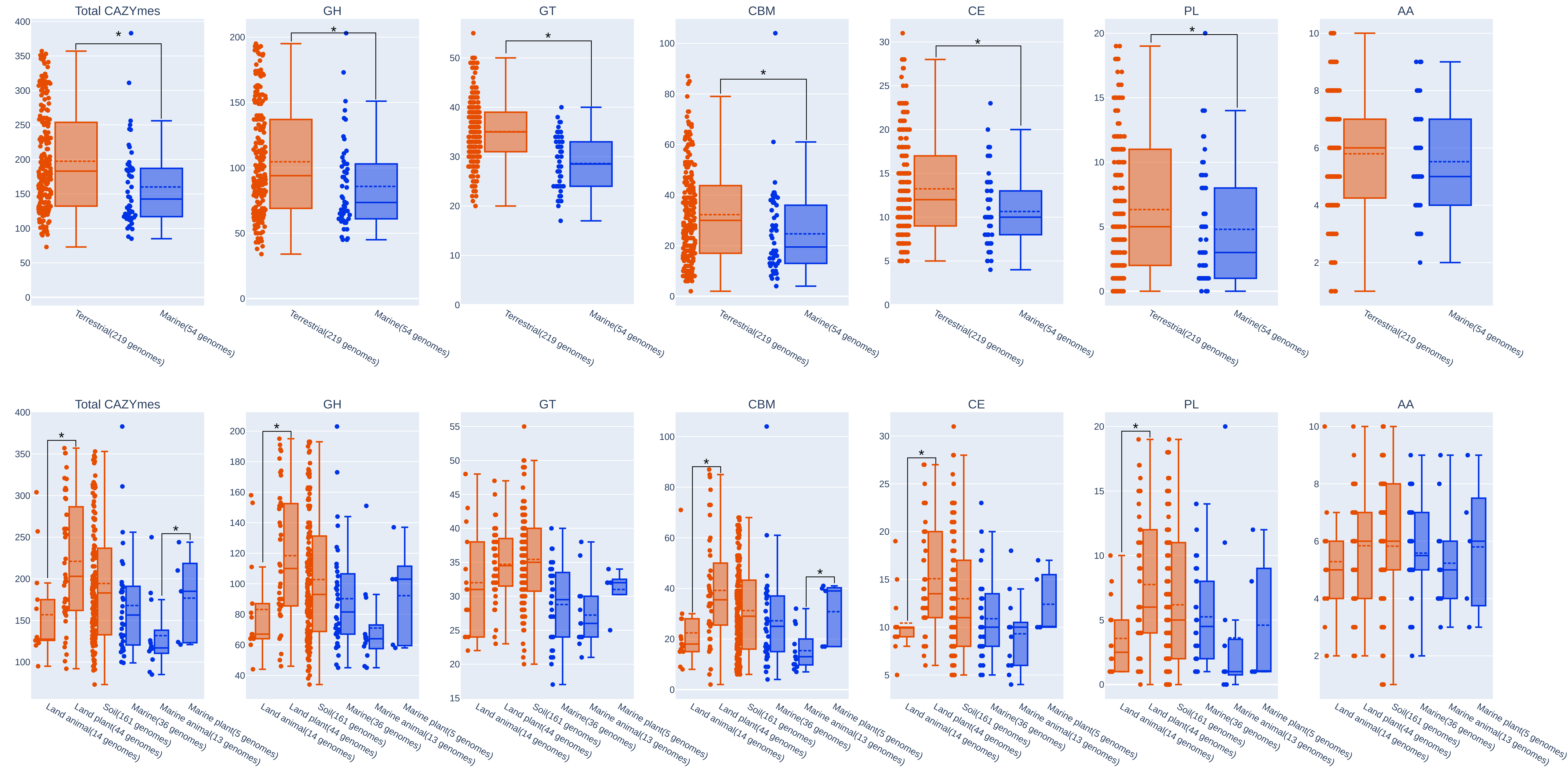

Supplement: Supplementary material 1 [file mgen-9-1028-s001.pdf]
